# *De Novo* Assembly and Characterization of Two Transcriptomes Reveal Multiple Light-Mediated Functions in the Scallop Eye (Bivalvia: Pectinidae)

**DOI:** 10.1371/journal.pone.0069852

**Published:** 2013-07-29

**Authors:** Autum N. Pairett, Jeanne M. Serb

**Affiliations:** Department of Ecology, Evolution, and Organismal Biology, Iowa State University, Ames, Iowa, United States of America; Karlsruhe Institute of Technology, Germany

## Abstract

**Background:**

The eye has evolved across 13 separate lineages of molluscs. Yet, there have been very few studies examining the molecular machinary underlying eye function of this group, which is due, in part, to a lack of genomic resources. The scallop (Bivalvia: Pectinidae) represents a compeling molluscan model to study photoreception due to its morphologically novel and separately evolved mirror-type eye. We sequenced the adult eye transcriptome of two scallop species to: 1) identify the phototransduction pathway components; 2) identify any additional light detection functions; and 3) test the hypothesis that molluscs possess genes not found in other animal lineages.

**Results:**

A total of 3,039 contigs from the bay scallop, *Argopecten irradians* and 26,395 contigs from the sea scallop, *Placopecten magellanicus* were produced by 454 sequencing. Targeted BLAST searches and functional annotation using Gene Ontology (GO) terms and KEGG pathways identified transcripts from three light detection systems: two phototransduction pathways and the circadian clock, a previously unrecognized function of the scallop eye. By comparing the scallop transcriptomes to molluscan and non-molluscan genomes, we discovered that a large proportion of the transcripts (7,776 sequences) may be specific to the scallop lineage. Nearly one-third of these contain transmembrane protein domains, suggesting these unannotated transcripts may be sensory receptors.

**Conclusions:**

Our data provide the most comprehensive transcriptomic resource currently available from a single molluscan eye type. Candidate genes potentially involved in sensory reception were identified, and are worthy of further investigation. This resource, combined with recent phylogenetic and genomic data, provides a strong foundation for future investigations of the function and evolution of molluscan photosensory systems in this morphologically and taxonomically diverse phylum.

## Introduction

The eye has long been a favorite system to study evolution. This is in part because image-forming eyes have evolved many times across separate animal lineages [1], [2] and functional components of the eye, like photoreceptor cells, their phototransduction genes, and lens proteins, may have different evolutionary histories (reviewed in [3]). Thus, eye evolution can be treated as a series of natural experiments to examine both molecular and physiological innovations, as well as a way to identify functional limitations. To fully understand how eye function changes over time, it is important to recognize the full suite of genes and their interacting genetic pathways involved in the functional phenotype across a diverse set of species. These data can then be applied to hypothesis-based research and experimental studies. In spite of this interest, eye studies have concentrated on only a few model organisms. Even with the advent of next-generation sequencing technologies and transcriptomic studies, only a fraction of the known eye diversity is represented in public databases [4], [5]. The majority of these genetic resources are from the camera type eyes of vertebrates and the compound eyes of arthropods, while the second largest animal phylum, the Mollusca, has been largely ignored (but see [6], [7]). Molluscs possess a wide range of eye diversity, representing four of the eight major eye types in animals [8]. Some examples of molluscan eye types include the pit eyes of some gastropods (e.g. limpets [9]), the basic compound eyes of ark clams [10], [11], the vertebrate-like camera type eye of coleoid cephalopods (e.g. *Octopus vulgaris* [12]), and the mirror-type eyes found in scallops [13] (other molluscan eye types reviewed in [14]–[17]). These morphologically divergent eye types have evolved in 13 separate lineages [18], with the majority of these occuring in the bivalves, the second largest molluscan class comprising over 30,000 extant species [19]. Thus molluscs, and more specifically bivalves, represent a rich, under-ulitized system to study eye evolution.

**Figure 1:**
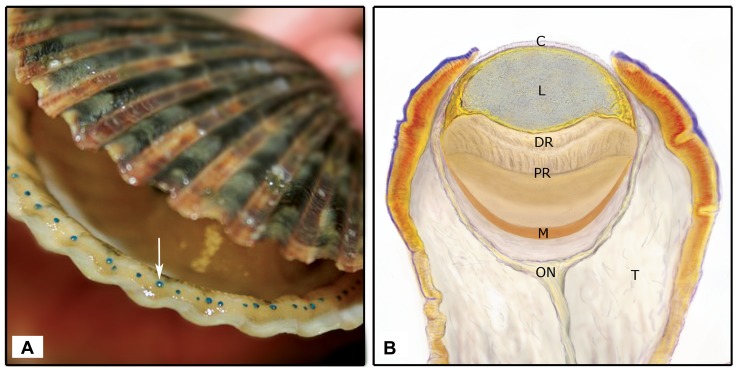
The mirror type eye of the scallop *Argopecten irradians.* (**A**) Scallops have up to 200 single chambered eyes along their mantle edge, indicated by the white arrow. (**B**) The eye sits on a tentacle ( =  T) and the major internal structures of the scallop eye are indicated. C =  cornea, L =  lens, DR =  distal retina, PR =  proximal retina, M =  mirror, ON = optic nerve.

One family of marine bivalves, the scallops (Pectindae), is of particular interest to vision research as it is a diverse family with a complex visual system that can construct a spatially resolved representation of the animal’s environment [20]. Each animal has between 30 and 200 non-cephalic eyes placed along the middle fold of the mantle lining the shell (Fig. 1A). The back of each single chambered eye is lined by a reflective argentea that focuses light on one of two stacked retinas ([21], [22]; Fig. 1B). The proximal retina contains rhabdomeric photoreceptor cells, characteristic of invertebrate eyes, and a corresponding r- or Gq-coupled, opsin [23]. The distal retina is composed of ciliary photoreceptor cells, with an opsin that pairs with a Go-protein (Go-coupled opsin; [23]), rather than the more common pairing of a ciliary opsin with a Gt-protein. Notably, both Go- and Gt-proteins are members of the same Gi-protein family [24].

The anatomy and physiology of the scallop eye has been studied extensively to determine the visual capabilities of this eye type (e.g., [13], [21], [22], [25]–[31]). More recently, workers have begun to identify the molecular components of scallop vision directly through sequencing [23], [32] or indirectly with phamacological targeting of putative pathway members [29]. However, gene membership of important light-sensing pathways, such as phototransduction, is still largely unknown, as are the genetic components underlying other neural functions (e.g. neuropeptide signaling). We sequenced the adult eye transcriptome of two scallop species, the bay scallop, *Argopecten irradians*, and the sea scallop, *Placopecten magellanicus*, to fill these gaps in knowledge. Both species are economically important to North American fisheries [33], [34] and as a result, have served as laboratory models for physiological [35]–[38] and developmental [39]–[42] studies. Our study had three goals: 1) to develop a more comprehensive understanding of gene membership of the two phototransduction pathways; 2) to determine if the scallop eye performs any other non-visual, light-mediated functions; and 3) to test the claim that molluscan genomes contain “unique” genes [43]–[45], not homologous to other metazoan taxa, by comparing our data to the newly available oyster (*Crassostrea gigas*, [45]), the limpet gastropod (*Lottia gigantea*, http://http://genome.jgi-psf.org/Lotgi1/Lotgi1.download.html), fruit fly (*Drosophila melanogaster*), and house mouse (*Mus musculus*) genomes.

**Figure 2:**
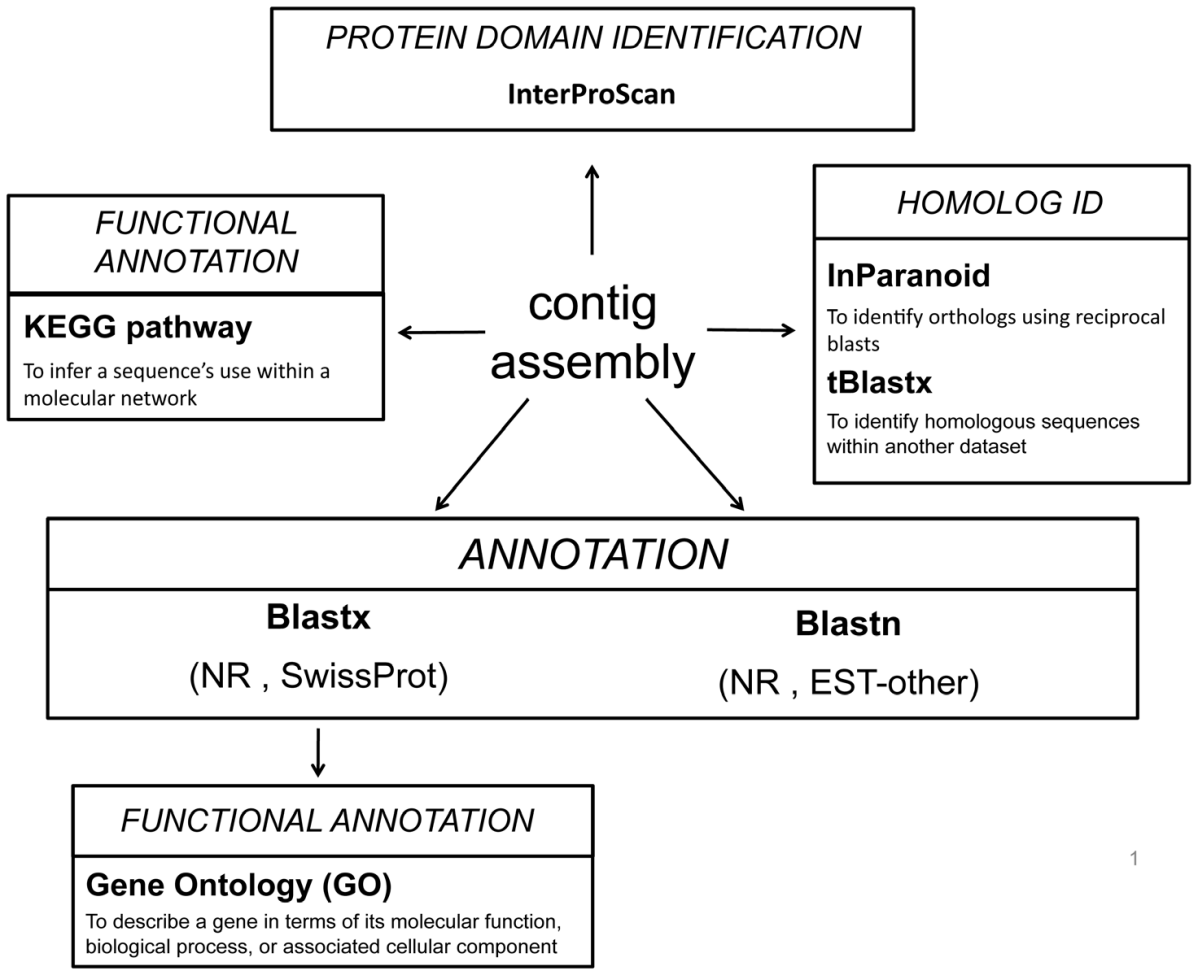
Sequence of analyses performed to assemble and annotate scallop eye transcriptomes.

Here we present 3,039 assembled sequences from the eye transcriptome of *A. irradians* and 26,395 assembled sequences from the *P. magellanicus* eye transcriptome, which were generated using 454 sequencing technology, then analyzed using a workflow developed to increase annotation success in a non-model system (Fig. 2). Based on the annotated suite of genes, the scallop eye has multiple light-mediated functions. In addition to containing the genetic components for two independent phototransduction pathways (Gq- and Go-protein mediated), our transcriptome analysis identified all six genes central to circadian clock function, indicating that the scallop eye is an important entry point for light entrainment of the clock. The scallop eye transcriptomes also contain a large number of putatively mollusc-specific, bivalve-specific, and scallop-specific genes that could not be annotated using BLAST algorithms. Many of these sequences were recognized as transmembrane regions and/or signal peptides, which suggest that the scallop sensory system may contain an array of currently undescribed sensory receptors. These data are important for future investigations on the molecular mechanisms that underlie photosensory systems of molluscs and fill a taxonomic gap in eye evolution.

## Results

### Transcriptome Sequencing and Assembly

A portion of the *Argopecten irradians* eye transcriptome was sequenced using both Sanger (1,920 reads, mean length of 821 bp) and 454 (112,132 reads, mean length of 348 bp) sequencing, while the *Placopecten magellanicus* transcriptome was generated using only 454 sequencing and produced 654,002 reads, averaging 273 bp in length (Table 1). Assembly of just the *A. irradians* 454 sequences produced 1,305 contigs with an N50 contig size of 697 bp and an average coverage of 20.4x. After trimming the datasets for vector sequences and *E. coli* contaminants, the two datasets from *A. irradians* were assembled together to form 3,039 contigs and singlets, with an average length of 529 bp (Table 1; GenBank dbEST accession numbers JK750414–JK752132 and GenBank TSA accession numbers JU170737–JU173620). Assembly of the *P. magellanicus* transcriptome created 34,964 contigs with an N50 contig size of 781 bp and an average coverage of 6.4x (Table 1). The assembled contigs were further grouped into 28,326 isotigs (representing individual transcripts) and 22,135 isogroups (representing genes) (GenBank TSA accession GADG00000000). The isotigs averaged 704 bp in length, with each isotig including an average of 1.9 contigs, and the isogroups averaged 1.3 isotigs per group. Only isotigs that were longer than 100 bp (26,395 sequences) were used in the next set of analyses.

**Table 1:** Summary of *A. irradians* and *P. magellanicus* eye transcriptome sequence and assembly data.

		A. irradians	P. magellanicus
**Raw Data**	Number reads	114,052^a^	654,002
	Average length	356 bp	273 bp
**Assemblies**	Number contigs+singlets	3,039	34,964^b^
	Average length	529.4 bp	463 bp
	N50 contig size	697 bp^c^	781 bp
	Average coverage	20.4^c^	6.4
**Annotations**	Sequences annotated by BLAST	1,962 (64.56%)	17,612 (66.7%)
	Sequences annotated with GO terms	857 (28.2%)	8,926 (33.8%)

a The *A. irradians* eye transcritome data came from a combination of Sanger and 454 sequencing, while the *P. magellnanicus* data were produced using 454 sequencing.

b Only those sequences that were greater than 100 bp were analyzed (26,395 sequences) in *P. magellanicus*.

c These data only apply to those sequences produced by 454 sequencing (112,132 sequences).

To examine the completeness of the *A. irradians* and *P. magellanicus* eye transcriptomes, we blasted each against a dataset of 458 core eukaryotic genes that are expected to be found in a wide range of species [46]. Of the 2,748 sequences in this dataset, 1,024 (37.26%) were identified in *A. irradians*, while 2,270 (82.6%) were found in the *P. magellanicus* eye transcriptome. The *P. magellanicus* transcriptome contains a high proportion of the core eukaryotic genes, suggesting that it is a good quality and representative dataset of genes expressed in the *P. magellanicus* eye. The *A. irradians* eye transcriptome, however, contains a much lower proportion of core eukaryotic genes. This indicates that this dataset does not reflect the whole *A. irradians* transcriptome, but a portion of it. To reflect this, we refer to the *A. irradians* transcriptome as the *A. irradians* dataset from this point on.

### Sequence Annotation Identifies Three Light Detection Pathways Within the Scallop Eye

**Figure 3:**
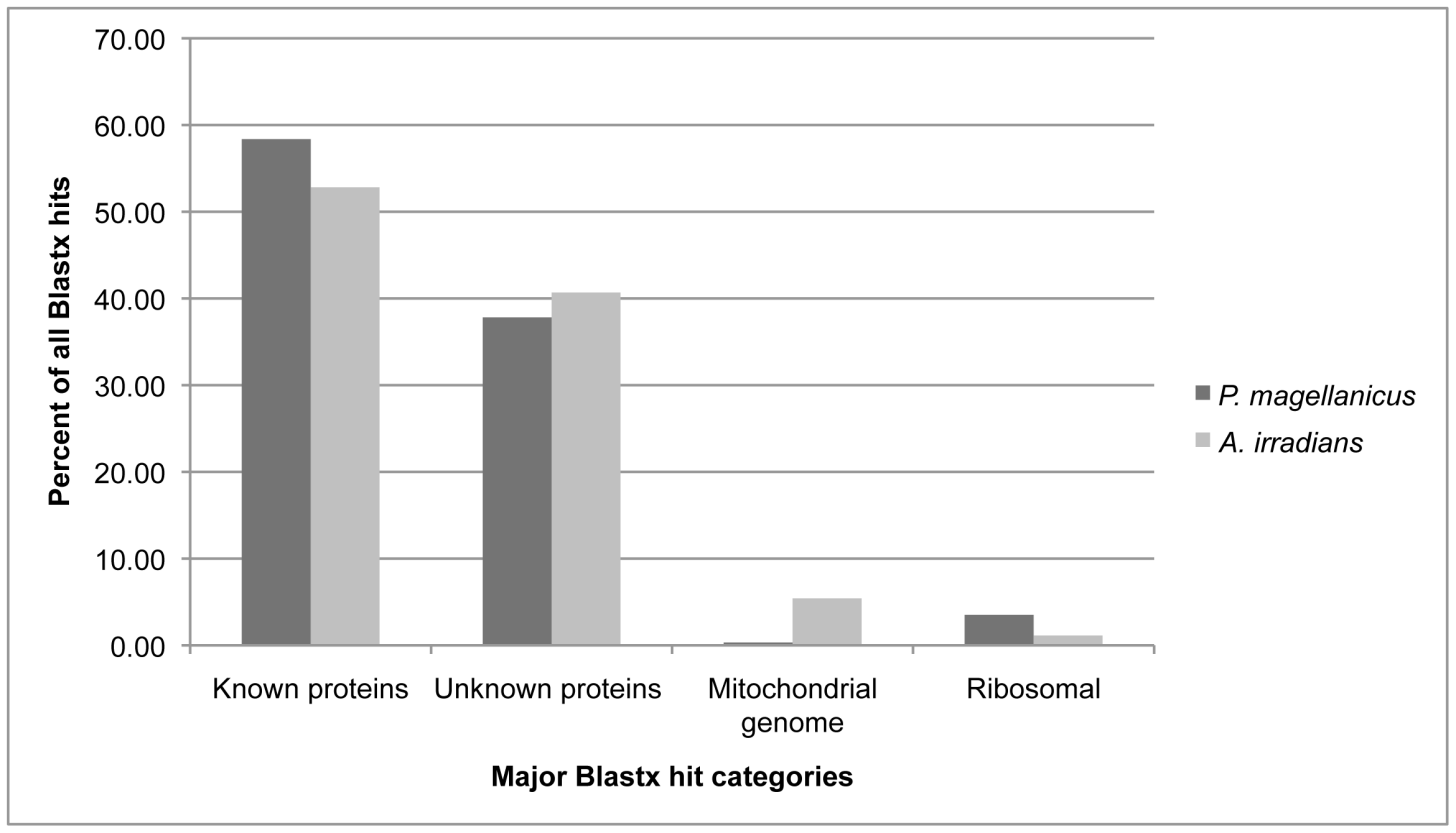
Scallop BLAST hit distributions. Percent hits in each major category of proteins examined (“Known” = annotated nuclear proteins; “Unknown” = proteins described as putative, hypothetical, unknown, or a species mRNA clone; “Mitochondrial genome” = annotated mitochondrial proteins; “Ribosomal” = ribosomal genes).

To annotate the scallop eye transcriptomes and identify genes related to light detection in the eye, we utilized several automated annotation methods (four BLAST schemes, Gene Ontology (GO) term searches, and Kyoto Encyclopedia of Genes and Genomes (KEGG) pathway analyses; Fig. 2) as well as targeted BLAST searches of the scallop transcriptomes for genes of interest. Using the four-part BLAST strategy, 64.56% (1,962 sequences) of the *A. irradians* dataset and 66.72% (17,612 sequences) of the *P. magellanicus* transcriptome were annotated (Table 1). Excluding ribosomal genes or genes from the mitochondrial genome, just over 50% of each dataset matched known proteins (*A. irradians*: 52.8%, 1,036 sequences; *P. magellanicus*: 58.37%, 10,280 sequences), while 40.67% (798 sequences) of the hits in *A. irradians* and 37.8% (6,657 sequences) of the hits in *P. magellanicus* came from unknown proteins or Genbank entries that are described as hypothetical, predicted, unknown, or a species clone (Fig. 3).

Targeted BLAST searches using a dataset of protein sequences from the phototransduction and circadian rhythm pathways (Table S1) identified most of the genes necessary for phototransduction as well as genes for circadian rhythm (amino acid alignments provided in Fig. S1–S3). Genes involved in the circadian clock have not been recognized in the scallop eye before this study. Not surprisingly, the *P*. *magellanicus* transcriptome, which has the greatest sequencing depth, contains a more complete representation of the genes from the two major types of phototransduction cascades described in the scallop eye (Gq- and Gi-protein mediated) and circadian rhythm pathway (Table 2). Of the 19 genes involved in the Gq phototransduction cascade, we found 16 genes in the *P. magellanicus* transcriptome, while the *A. irradians* dataset only had eight. Similarly, for the Gi phototransduction cascade, which includes both the Go- and Gt-protein mediated phototransduction cascades, the *P. magellanicus* eye transcriptome contains 12 of the 16 described genes, while seven of the 16 were found in the *A. irradians* dataset (Table 2). Five of the six core circadian clock genes described from *M. musculus* and *D. melanogaster* were identified in the scallop eye transcriptomes using KEGG pathway analysis (Table 2, Table S4), but *period* was not found with this approach. We then applied a targeted BLAST strategy using homologs from *D. melanogaster*, *M. musculus*, and three molluscs: a gastropod (*Bulla gouldiana*) and two bivalves (*Mytilus galloprovincialis* and *Crassostrea gigas*) to identify the *period* gene. Only the *C. gigas period* homolog recovered a significant, but high E-value, hit in the *P. magellanicus* eye transcriptome. This was surprising as antibodies against the *Drosophila* homolog have been used to visualize the protein expression pattern of *period* in two gastropod species (*Aplysia californica* and *Bulla gouldiana*; [47]). A comparison of *period* homologs between *Drosophila* and *B. gouldiana* found low conservation of amino acid sequence (20.7% identical in amino acid sequence). This is a consistent pattern when comparing *period* protein homologs between *Drosophila* versus *C. gigas* (20.4% identical in amino acid sequence) and *B. gouldiana* versus *C. gigas* (26.2% identical in amino acid sequence) or between two oyster species (*C. gigas* versus *Pinctada fucata*, 41.9% identical in amino acid sequence ). These data suggest that *period* is a highly divergent gene, making it difficult to identify using traditional automated annotation methods in molluscs.

**Table 2:** Presence of key phototransduction and circadian clock genes in scallop eye transcriptomes.

Pathway		Pathway Gene Name	P. magellanicus	A. irradians
Phototransduction	Gq	calmodulin	B, G, K	B, K
		CD synthase (cdsA)		
		DAG kinase (rdgA)	B, G	B
		Gq-protein alpha subunit	B, K	B
		inaD		
		ninaC (myosin III)		B
		phospholipase C (norpA)	B, G, K	
		PI kinase	B	
		PI synthase	B	
		PI transferase (rdgB)	B	
		PIP kinase	B, G	B
		protein kinase C (inaC)	B, G, K	
		TRP	B	
		TRPL	B	
	Gi	cGMP gated channel alpha	B	
		cGMP gated channel beta	B	
		Go-protein alpha subuint	B, G	B
		Gt-protein alpha subunit	B	B
		guanyl cyclase		
		PDE alpha	B	
		PDE beta	B	
		PDE gamma		
		phosducin		
		protein kinase A	B	B
		recoverin		B
	Both Gq/Gi	arrestin	B, G, K	
		G-protein beta subunit	B, G, K	B
		G-protein gamma subunit	B, G	
		opsin	B, G	B, K
		rhodopsin kinase	B	B
Circadian clock		clock	B, K	
		cryptochrome	B, K	
		cycle	B, K	
		doubletime	B, K	
		period	B	
		timeless	B, K	

Gene presence is indicated by the letters *B*, *G*, or *K*, which describes method of identification (*B* = BLAST, *G* = GO terms, *K* = KEGG pathway analysis).

**Figure 4:**
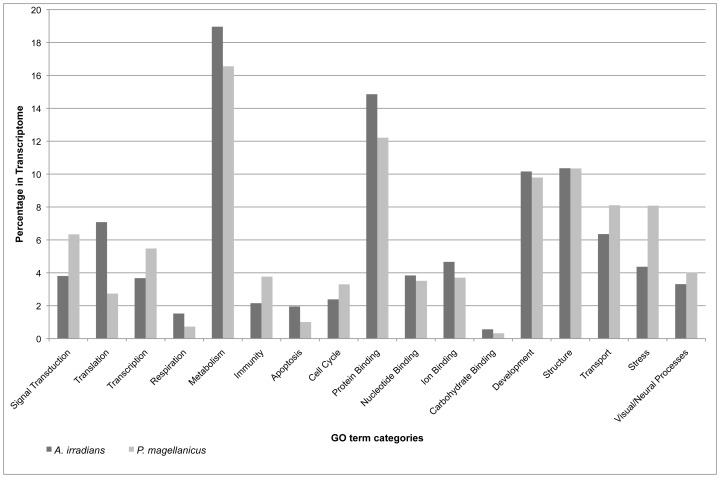
Distribution of Gene Ontology (GO) term categories in *A.* *irradians* and *P. magellancius* eye transcriptomes. Bars show the percent of sequences with a GO term annotation for each category.

From the GO term analyses, we found that vision and neural processes GO terms comprised 3% of the *A. irradians* dataset (Fig. 4, Table S2) and 4% of the *P. magellanicus* eye transcriptome (Fig. 4, Table S3). The majority of GO terms in both scallop eye datasets are related to metabolism (19% in *A. irradians,* 17% in *P. magellanicus*), protein binding (15% in *A. irradians,* 12% in *P. magellanicus*), structure (10% in both) and development (10% in both; Fig. 4). Most of the sequences with visual or neural process related GO term annotations were associated with neural processes (51 in *A. irradians*; 1,582 in *P. magellanicus*), while few sequences were labeled with vision-related GO terms (13 in *A. irradians* and 62 in *P. magellanicus*; Tables S2 and S3). Most neural process GO terms were related to neurogenesis or neuron differentiation and development (24 sequences in *A. irradians*; 1,028 sequences in *P. magellanicus*), with the remaining GO terms generally being related to terms such as neurological system process (286 in *P. magellanicus*), neurotransmitter activity or synapses (12 in *A. irradians* and 97 in *P. magellanicus*), and neuron cell parts or action potentials (15 in *A. irradians*, 162 in *P. magellanicus*). GO term annotation also identified two key neuropeptide genes in *P. magellanicus*, *neuropeptide y* and the *neuropeptide y receptor type 6*, which have been shown to be necessary for circadian rhythm entrainment in mouse [48]. All of the GO terms related to vision were specific to visual perception, while the retina-related GO term category consisted of terms like retina development, retinal binding, and retinal dehydrogenase activity.

Only a few genes from the phototransduction pathways were identified with GO annotation (Table 2; Tables S2 and S3). A similar pattern was found with the KEGG pathway analysis, which identified just four phototransduction genes in *A. irradians* and six phototransduction genes in *P. magellanicus* (Table 2, Table S4). *Opsin* was not identified in *P. magellanicus* using KEGG, even though two Gq-coupled opsin sequences were found using BLAST and GO terms. Interestingly, while the KEGG pathway analysis did not identify many of phototransduction genes, five of the six core members of the circadian rhythm pathway (*timeless*, *clock*, *doubltime*, *cycle,* and *cryptochrome*) were identified in the *P. magellanicus* eye transcriptome using KEGG (Table 2, Table S4).

### Protein Domain Annotation Identifies Many Transmembrane Proteins

Due to the large number of unannotated sequences and unknown proteins from our BLAST analyses, we attempted to classify these sequences by protein domain annotation with InterProScan (Table 3). For the scallop eye transcriptomes, 49.16 (*A. irradians*) to 54.97% (*P. magellanicus*) of the sequences were annotated with protein domains. On average, 2.5 protein domains were annotated per sequence for a total of 3,463 and 38,778 annotated protein domains for *A. irradians* and *P. magellanicus*, respectively. Of the sequences annotated by InterProScan, about 38% had no previous blastx annotation, representing 19.12% of the *A. irradians* dataset and 20.24% of the *P. magellanicus* transcriptome.

**Table 3:** Protein domain annotation of *A. irradians* and *P. magellanicus* adult eye transcriptomes.

	A. irradians			P. magellanicus		
	*# seq*	*% transcriptome*	*% InterProScan hits*	*# seq*	*% transcriptome*	*% InterProScan hits*
Transcriptome size	3,039			26,395		
InterProScan hits	1,494	49.16		14,509	54.97	
Annotated by blastx	1,243	40.90		11,814	44.76	
Annotated by blastx and InterProScan	913	30.04	61.11	9,165	34.72	63.17
Annotated by InterProScan only	581	19.12	38.89	5,344	20.25	36.83
Number of protein domains annotated	3,463			38,778		
Average protein domainsannotated per sequence	2.32			2.67		
Number of putative genes (isogroups)	N/A^a^			22,135		
Number of putative genes (homology)^b^	919			4,960		

a Putative genes are based on isogroups constructed during the assembly of the transcriptome. Because the *A. irradians* dataset was constructed from a combination of Sanger and 454 sequences, isogroups were not calculated for these data.

b Putative genes based on homology were identified by blastx blasts to the predicted gene models of the *Lottia gigantea* genome (E-value cutoff of E-3.

The most abundant protein domain annotations in both transcriptomes were transmembrane regions (present in 35.81% of the annotated sequences from *A. irradians*, and in 34.09% of the *P. magellanicus* annotated sequences) and signal peptides (42.17% and 39.84% of the annotated sequences in *A. irradians* and *P. magellanicus* datasets, respectively; Table 4). Two additional protein domain categories were examined: G-protein coupled receptors (GPCRs), due to their prevalance in sensory systems, and transcription factors. A very small proportion of annotated sequences were GPCRs in each scallop eye dataset (0.4% and 0.3% in *A. irradians* and *P. magellanicus*, respectively). One sequence was annotated as “opsin” in each scallop eye transcriptome, while the remaining annotated GPCRs were identified as “Family A” GPCRs or simply “G-protein receptors.” Transcription factors, on the other hand, made up a larger proportion of the InterProScan annotated sequences. Seven types of transcription factors were identified in *P. magellanicus* (paired box, homeodomain, winged helix, zinc finger, helix-turn-helix, bzip, and srf-like), while the *A. irradians* dataset only contained representatives from three groups of transcription factors (homeodomain, bzip, and winged helix). Overall, most of the transcription factors identified were zinc fingers, while homeodomains were more common in the *P. magellancius* transcriptome than in the *A. irradians* dataset (Table 4).

**Table 4:** Major categories of protein domains identified by InterProScan for *A. irradians* and *P. magellanicus* adult eye transcriptomes.

	A. irradians		P. magellanicus	
	*# sequences*	*% InterProScan hits*	*# sequences*	*% InterProScan hits*
Ribosomal	86	5.76	411	2.83
Transmembrane regions	535	35.81	4946	34.09
Signal peptides	630	42.17	5780	39.84
GPCRs	6	0.40	44	0.30
Transcription factors - all	41	2.74	538	3.71
Transcription factors - Zinc fingers	34	2.28	388	2.67
Transcription factors - Homeodomains	1	0.07	43	0.30
Transcription factors - Others	8	0.54	138	0.95

### Homolog Identification Against Large Molluscan and non-molluscan Genetic Datasets Reveal Putative Scallop-, bivalve-, and Mollusc- specific Genes

To identify homologous genes between the two scallop eye transcriptomes and to identify putatively scallop-specific sequences, we first blasted each scallop eye dataset to the other using tblastx with an E-value cutoff of E-3 (*A. irradians* vs. *P. magellanicus* and *P. magellancius* vs. *A. irradians*). When blasting the *A. irradians* adult eye dataset against the *P. magellanicus* adult eye transcriptome (*A. irradians* = query, *P. magellanicus* = subject), 1,096 sequences (36.06% of the *A. irradians* dataset) had significant hits. About 43% of those (470 sequences) had no matches in the NCBI databases. The reciprocal analysis (*P. magellanicus* = query, *A. irradians* = subject) produced a total of 3,449 significant hits (13.07% of the *P. magellanicus* transcriptome). Only 22.67% of the significant hits from this analysis (782 sequences) were not previously annotated by BLAST.

**Figure 5:**
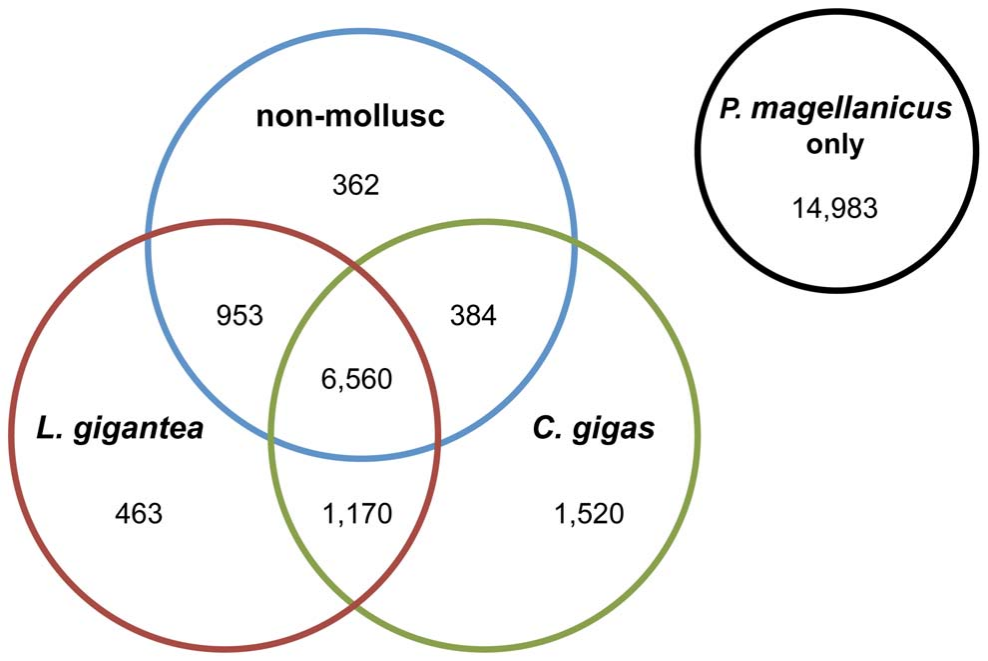
Venn diagram of *P.* *magellanicus* transcriptome sequences with significant blast hits against other animal genomes. The labels in each circle represent the animal genomes the *P. magellanicus* eye transcriptome was blasted against: the pacific oyster, *Crassostrea gigas* (green), the owl limpet, *Lottia gigantea* (red), and two non-mollusc genomes, *Drosophila melanogaster* and *Mus musculus* (blue). The number of sequences from *P. magellanicus* that did not match any other animal genome and are putatively unique to scallops are shown in a separate circle (black).

In order to identify potential mollusc-, bivalve-, and scallop-specific sequences, we compared our most comprehensive scallop eye transcriptome (*P. magellanicus*) against available molluscan and non-molluscan genome sequences, including the owl limpet *Lottia gigantea,* the pacific oyster *Crassostrea gigas* [45], the fruit fly *Drosophila melanogaster*, and the house mouse *Mus musculus* (Fig. 5). BLAST searches of *P. magellanicus* against the *L. gigantea* genome produced 9,146 significant hits, representing 34.65% of the scallop eye transcriptome. Blasts against the *C. gigas* genome had a similar number of significant hits (9,634 sequences or 36.5% of the transcriptome). We then conducted a BLAST search of the *P. magellanicus* transcriptome against predicted gene models from both *D. melanogaster* and *M. musculus* genomes, which returned a total of 8,259 hits. When we compared these results to those from blasts to the *L. gigantea* and *C. gigas* genomes, we found that 3,153 *P. magellanicus* sequences only matched the molluscan genomes and likely represent putative mollusc-specific genes. Of these 3,153 putatively mollusc-specific sequences, nearly half (1,520) correspond to regions of the *C. gigas* genome, but not *L. gigantea*, and are potentially bivalve-specific genes (Fig. 5). Overall, 14,983 *P. magellanicus* sequences did not match any of the genomes examined, with 7,776 of those returning no significant results (E-value cutoff of E-3), even after applying our four-part BLAST strategy (described in Fig. 2). To determine if low hit return was due to low sequence quality, we examined the lengths of the 7,776 sequences. These sequences ranged in length from 100–2,541 bp (mean = 637 bp), where 2,475 reads (31.8%) were between 200–499 bp, 4,136 reads (53.2%) were between 500–999 bp, and 806 reads (10.4%) were 1,000 bp or more. Thus, the lack of BLAST hits are not due to poor sequence quality. Rather, these unannotated sequences may represent reads that are specific to the scallop lineage.

Additional blasts of both scallop eye datasets were performed against ESTs from the central nervous system (CNS) of the great pond snail *Lymnaea stagnalis* [44] and the eye transcriptome of the common octopus *Octopus vulgaris* [6] to identify genes commonly expressed between molluscan nervous systems and eyes. Only 398 sequences matched between *L. stagnalis* and *A. irradians* (13.09% of the dataset), while 3,749 sequences from *P. magellanicus* had a significant hit to the *L. stagnalis* ESTs (14.02% of the transcriptome). Blasts to the octopus eye dataset had similar results, with just 304 sequences (10% of the *A. irradians* dataset) matching between *A. irradians* and octopus, while 2,319 sequences (8.79% of the *P. magellanicus* transcriptome) were identified between *P. magellanicus* and *O. vulgaris*. In both analyses, most matching sequences had been annotated by BLAST previously. Only 19 (4.77%) and 93 (2.48%) of the sequences identified in *A. irradians* and *P. magellanicus*, respectively, using the *L. stagnalis* CNS ESTs were not previously annotated by BLAST. In the octopus comparison, 14 sequences in *A. irradians* (4.6% of the hits) and 106 sequences (4.57% of the hits) in *P. magellanicus* did not have a previous BLAST hit.

Finally, we performed pairwise reciprocal blasts between and within each dataset to 1) identify orthologs between the two datasets while 2) eliminating paralogous sequences. Putative orthologs were identified by comparing the two scallop eye transcriptomes to each other, to the predicted gene models from *L. gigantea*, to the CNS ESTs of *L. stagnalis* [44], and to the eye transcriptome from *O. vulgaris* [6] using the program InParanoid [49], [50]. InParanoid identified 273 orthologs between *A. irradians* and *P. magellanicus*. When the required percent overlap of matching sequences was reduced from the default value of 50% to 25%, the number of orthologs identified between the two scallop eye transcriptomes increased to 671. When compared to the *L. gigantea* genome, we found 557 orthologs in the *A. irradians* eye dataset, while only 14 were identified in *P. magellanicus*. Reducing the required percent overlap of matching sequences to 25% did not change the number of orthologs identified for this analysis. An InParanoid search for orthologs between the scallop eye datasets and the *L. stagnalis* CNS ESTs did not return any orthologous gene sequences. Lastly, comparisons to the octopus eye transcriptome using the 50% overlap requirement found 26 orthologs in *A. irradians* and 95 orthologs in *P. magellanicus,* while a 25% overlap criterion identified 94 ortholgs in *A. irradians* and 249 orthologs in *P. magellanicus.*

## Discussion

Here we present two transcriptome datasets from the mirror-type eye of the scallop species, *Argopecten irradians* and *Placopecten magellanicus*. Together, these data represent the largest dataset of any eye type within the phylum Mollusca. Our analyses show that these datasets contain a large percentage of currently unidentified or novel sequences, suggesting that continued sequencing efforts, followed by comparative studies and functional analyses, will be important to fully understand the genetic components of the visual systems in scallop and other molluscs.

### Dual Functionality of the Scallop Eye

In this study, one major goal was to identify the genetic components important for light detection within the scallop eye. We utilized a series of analyses meant to annotate and assign putative function to the scallop eye transcriptome sequences (Fig. 2), by which we confirmed the presence of two previously identified phototransduction pathways: 1) a Gq-protein coupled (Gq) phototransduction pathway and 2) a phototransduction pathway regulated by the Gi-protein family (which includes both Go- and Gt-proteins) [23], [29]. In addition, we have evidence for a circadian clock in the scallop eye as the transcriptome includes all six core circadian clock genes described in *M. musculus* and *D*. *melanogaster* (Table 2, Table S4), as well as a key neuropeptide (*neuropeptide y*) for circadian rhythm entrainment [39]. This is the first study to identify these circadian clock genes in scallop. These results point to a dual light detection function of the scallop eye: vision and entrainment of the circadian clock. Previous work in two gastropods species (*Bulla gouldiana* [42], *Aplysia californica* [51]) identified a circadian pacemaker within the eye. In *B. gouldiana*, the pacemaker is a small group of neurons that form a cone-shaped structure at the back of the eye and are physically and functionally separate from the photoreceptor cells of the retina [47], [52]. While in *A. californica*, the pacemaker neurons are secondary neurons in the outer layer of the retina [47], [53] that receive light information from photoreceptors within the pigment layer [53], [54] and extend into the optic nerve [47], [55]. Our work supports previous studies demonstrating the important link between the eye and the circadian clock in molluscs; however, while the spatial arrangement of the phototransduction pathways utilized by the scallop eye has been described [23], it is unknown which scallop tissues are involved in entraining the circadian clock. As the scallop eye is lined with an argentea composed of layers of guanine crystals that reflect, rather than absorb, light [26], it is unlikely that the scallop circadian pacemaker is located along the back of the eye, like in *B. gouldiana* and *A. californica*. Instead, the scallop circadian clock may be entrained by different classes of photoreceptor cells (as is the case for many vertebrates, reviewed in [56]) in one (or both) of the scallop retinas, or in tissues surrounding the eye, such as the eye stalk and/or optic nerve. Since extraoccular tissues are difficult to remove from the scallop eye during dissection, a small amount of these tissues were likely included in our sample. Thus, extraoccular tissue, such as the eye stalk, cannot be ruled out as a location for the circadian clock. An examination of protein expression patterns will be required to identify the specific tissue containing the scallop circadian clock.

Most of the phototransduction genes and core circadian clock genes were identified in the *P. magellanicus* eye transcriptome, most likely due to its greater sequencing depth. Just as sequencing depth seems to affect the light detection-related genes we were able to identify, the analyses performed also affected which genes of interest were found. The majority of the phototransduction genes were found using a targeted BLAST approach, as opposed to GO term or KEGG pathway analyses, while most the circadian rhythm genes were identified using KEGG pathways (with *period* being the only exception; Table 2). In fact, each analysis tended to identify different components of the phototransduction pathway. This might be attributed to the independently curated databases of GO and KEGG. In general, these databases are more limited in their representation of non-model species, thus restricting the methods’ ability to annotate a query sequence. Our results highlight the importance of utilizing multiple analysis tools in order to identify genes of interest within a large sequence dataset, especially in non-model systems, as using a single tool may leave interesting aspects of the data undiscovered.

### Novel Sequences

Our analyses show that a large proportion of the scallop eye transcriptome is composed of sequences that cannot be identified through various homology searches using publicly available sequence datasets. This pattern is not unique to our data, as similar proportions of unknown sequences have been found in other molluscan transcriptome studies [44], [57], [58]. As a result, some have suggested that mollusc genomes contain a set of genes particular to the phylum [43]–[45]. Even when comparing our most comprehensive transcriptome (*P. magellanicus*) against available molluscan genomes and two non-molluscan genomes, we found a large number of putatively bivalve-specific and mollusc-specific genes (Fig. 5). Further, we identified 7,776 sequences that may be unique to scallops and important for various aspects of scallop biology. Alternatively, these sequences may be evolving so rapidly within molluscs, or just the scallop lineage, that homology searches fail, despite our use of several different annotation methods (Fig. 2). Yet, 2,755 of these putatively novel scallop sequences were annotated as proteins with transmembrane regions and/or signal peptides. This is an intriguing pattern as signal peptides are necessary to incorporate proteins into cellular membranes or other organelles, while receptors for extracellular signals are often transmembrane proteins, such as G-protein coupled receptors (GPCRs). Work on the California sea hare *Aplysia californica* [59] and other animals (reviewed in [60], [61]) have shown that sensory systems, such as those for olfaction or gustation, utilize GPCRs that are highly divergent, even between closely related groups, which makes the identificaiton of these receptors difficult. The large number of previously unidentified transmembrane regions and signal peptides points to the possibility of our transcriptomes containing a high proportion of unidentified protein receptors which may be important to the scallop sensory system. Blasts of our scallop eye transcriptomes against an EST dataset of mantle tissue from the Yesso scallop, *Mizuhopecten yessoensis*, (GenBank dEST GH735679–GH736789, GR867665–GR868079) support the possibility that some of our gene sequences may originate from extraoccular sources; however, these represent a small portion of each eye transcriptome. No more than 10% of each eye dataset (*A. irradians*: 10.2%; *P. magellanicus*: 7.2%) had hits against the mantle ESTs (E-value cutoff of E-3). Therefore, these unidentified putative protein receptors may represent compontents of the chemosensory or mechanosensory systems found in scallop eye stalk (a modified tentacle). Additional work on these putative transmembrane proteins will be required to determine how they function within the scallop eye or other sensory structures.

### Conclusions

Mollusca, a phylum rich in functional and morphological eye diversity, is the next taxonomic frontier in eye evolution research. Technological advances in high-throughput sequencing have made previously inaccessible visual systems attainable for study. Here, we generated the adult eye transcriptome from two scallop species, which together represent the largest transcriptome dataset available for a single mollscan eye type. These data reveal not only the presence of two phototransduction pathways, but also a third light detection pathway, the circadian clock, which was previously unrecognized in scallops. In addition, we identify a large number of putatively novel transmembrane protein domains that may play a role in the scallop sensory system and are worthy of further investigation. These data, in conjunction with the recent availability of genomic [45] and phylogenetic resources [62], [63], have positioned molluscs as an important model system to unravel how visual systems and non-visual light detection systems arose and evolved within this morphologically diverse and specious phylum.

## Methods

### Tissue Collection, RNA Extaction, and Transcriptome Sequencing

#### Argopecten irradians

Forty-eight live individuals of *A. irradians* were purchased from the online seafood retailer, Farm-2-Market.com in May 2008. Upon receipt of the scallops, individuals were immediately removed from their shells and flash frozen with liquid nitrogen. Approximately 1g of eye tissue was dissected and pooled from the scallop mantles under a stereomicroscope. To reduce the amount of non-occular tissue, eyes were removed from the eyestalk (a modified sensory tentacle) by severing the eyestalk just under the base of the eye using fine tweezers. Eye tissues were then stored at -80°C. Tissue was shipped to Agencourt Bioscience Corporation for RNA isolation and normalized cDNA library construction. From the normalized library, 1,920 clones were sequenced by the Sanger method. A second round of sequencing using 454 chemistry was performed on the same normalized library. To reduce the number of cloning vector reads, inserts needed to be released from the plasmids. To do so, we grew ten library cultures by adding between 25µ L and 75µ L of the normalized cDNA library to 25 mL of LB+carbenicillin media and incubated at 37°C for 16 hrs (220 rpm). From each culture, we produced two plasmid preparations from 6 mL of culture using the QIAprep Spin MiniPrep kit (Qiagen, cat# 27106). All subsequent enzyme digestions were performed as directed by the manufacturer (New England Biolabs, Inc.), except 20 units of enzyme were used in a 50µ L reaction to digest approximately 2µ g of plasmid DNA. Reactions were incubated at the appropriate temperature for at least two hours. Plasmids were first digested by one of three enzymes (RsrII, EcoRI, or SmaI), followed by phenol:chloroform extraction and ethanol precipitation of the insert DNA. DNA was resuspended in 25µL dH_2_0 and DNA quantity and quality was determined using a spectrophotometer (NanoDrop 2000). To complete the excision of the cDNA insert, a second restriction digest using XhoI was performed on all samples, as described above. A total of 160 double digested samples were pooled and sent to the Center for Genomics and Bioinformatics at Indiana University Bloomington for gel extraction of the cDNA inserts and subsequent 454 sequencing. The library preparation was sequenced on one quarter of a plate on the 454 GS-FLX with Titanium chemistries. Resulting sequences were assembled using the Newbler *de novo* assembly program (v. 2.0.01.14; Roche). The *A. irradians* sequences resulting from Sanger sequencing have been deposited at DDBJ/EMBL/GenBank in the Expressed Sequence Tag database under accession numbers JK750414–JK752132. The assembled 454 and Sanger sequences are deposited under the Transcriptome Shotgun Assembly accession numbers JU170737–JU173620.

#### Placopecten magellanicus

Live *P. magellanicus* individuals were ordered from Farm-2-Market.com in February 2009. Upon arrival, scallops were placed in a saltwater tank and exposed to light for two hours. Mantle tissue from dissected animals was frozen at -80°C prior to eye dissection. Eyes from one individual were removed, as described above for *A. irradians* and the RNA extracted using the RiboPure RNA extraction kit (Ambion, part #AM1924) following the manufacturer’s instructions. Extracted RNA was then cleaned using a standard chloroform extraction protocol. Briefly, an equal volume of chloroform was added to the extracted RNA, shaken to create a homogeneous solution, and centrifuged at 4°C for 15 minutes to separate the organic and aqueous layers. The aqueous layer was removed, then 1/10 volume of sodium acetate (3M, pH = 5.2) and 2.5 volumes of 100% ethanol were added. RNA was then stored overnight at -20°C. The RNA was precipitated by spinning at 4°C for 15 minutes. The pellet was washed with 70% ethanol and spun for 10 minutes to remove ethanol. The RNA pellet was air dried, then resuspended in RNase free water. Extracted and cleaned RNA was sent to the Center for Genomics and Bioinformatics at Indiana University Bloomington for normalized cDNA library construction and 454 sequencing. The normalized cDNA library was sequenced on one half plate of the 454 GS-FLX with Titanium chemistries. Resulting sequences were assembled using the Newbler *de novo* assembly program (v. 2.3; Roche). The *P. magellanicus* Transcriptome Shotgun Assembly project has been deposited at DDBJ/EMBL/GenBank under the accession GADG00000000. The version described in this paper is the first version, GADG01000000.

### Transcriptome Annotation

In *Argopecten irradians*, 454 sequencing produced 112,132 reads, which were assembled into 3,495 contigs. Vector sequences were trimmed through the program Geneious (v. 4.7; Biomatters, available from http://www.geneious.com) and manually checked before being removed from the contigs, leaving 3,039 sequences. Sequencing of the *Placopecten magellanicus* eye transcriptome using 454 techonology resulted in 654,002 reads, which were assembled into 34,964 contigs. Transcriptome sequence assembly by the Center for Genomics and Bioinformatics (CGB) at the University of Indiana, Bloomington produced 28,326 isotigs, or sequences representing individual transcripts. Only those sequences that were more than 100 bp were used in further analyses (26,395 sequences). Transcriptome completeness was examined by blasting each transcriptome against a dataset of 458 core eukaryotic genes [46] using tblastn and an E-value cutoff of E-3. A dataset of mantle tissue ESTs from the Yesso scallop, *Mizuhopecten yessoensis*, was downloaded from GenBank (dbEST GH735679–GH736789, GR867665–GR868079) and blasted against each scallop eye dataset to examine the extent of possible mantle tissue contamination.

For both datasets, sequences were subjected to a series of annotation programs (Fig. 2). First, we applied a four-part blast strategy which uses two different BLAST tools against different publically available databases: 1) blastx with the NCBI (National Center for Biotechnology Information) non-redundant (nr) database, 2) blastx with the SwissProt database, 3) blastn with the NCBI nr database, and 4) blastn with the NCBI est_others database. Each search applied an E-value cutoff of E-3 to maximize the number of sequences annotated by BLAST. All BLAST outputs were combined, then the best annotation was chosen based on the lowest E-value and the most descriptive annotation (e.g. for one sequence, the blastn output is *“A. irradians* full mitochondrial genome” while the blastx output is “cytochrome oxidase I.” Cytochrome oxidase I would be chosen as the sequence annotation.) Annotated contaminant sequences (e.g. *E. coli*) were checked manually with additional blastx and blastn searches in Geneious, then removed from further analysis. Second, sequence annotations from blastx searches were used for subsequent Gene Ontology (GO) term mapping in Blast2GO [64] using the default settings. To determine the distribution of different categories of GO terms, the resulting GO term annotations were sorted by key words, such as translation, transcription, protein binding, and structure. Third, sequences from the contig assembly were subjected to Kyoto Encyclopedia of Genes and Genomes (KEGG) pathway annotation using the KEGG Automatic Annotation Server (KAAS) (www.genome.jp/tools/kaas/). Results from the analysis were visualized using the program KegHier (available from http://www.kegg.jp/kegg/download/kegtools.html). Last, assembled transcriptome sequences were annotated with protein domains using the InterProScan program plugin for Geneious (v. 5.5; Biomatters, available from http://www.geneious.com). The InterProScan searches were performed using default settings against all available protein domain databases.

### Homolog Identification Using BLAST and InParanoid

To identify homologous gene sequences within the two scallop eye transcriptomes, we ran two types of analyses (Fig. 2). First we sought to identify homologous sequences between the scallop eye transcriptomes, as well as between the scallop datasets and other molluscan and non-molluscan genetic datasets. Therefore, we performed BLAST searches between each scallop eye transcriptome, two available mollusc genomes (*C. gigas,* [45] and *L. gigantea,* http://genome.jgi-psf.org/Lotgi1/Lotgi1.download.html), a dataset of snail central nervous system ESTs (*L. stagnalis*, [44]), the octopus eye transcriptome (*O. vulgaris*, [6]), predicted genes from the *D. melanogaste*r genome, and predicted genes from the *M. musculus* genome using the tblastx algorithm and an E-value cutoff of E-3 in the stand-alone version of BLAST (v. 2.2.23, NCBI). Second, we took a targeted BLAST approach in order to identify key genes from the phototransduction pathway and the circadian clock within each scallop eye transcriptome by downloading protein sequences for the genes from each pathway from NCBI, then blasting them against each scallop eye transcriptome in the program Geneious (v. 5.5, Biomatters). Since the interaction between a Go-protein and cilary opsin is uncommon in metazoans, we used phototransduction genes from the more inclusive Gi-protein family, which includes both Go- and Gt-proteins [24]. Gene names and accession numbers are in Table S1. We confirmed the identity of scallop sequences that hit to one of the light detection pathway genes with an E-value less than E-3 in two ways. First, these sequences were blasted against the NCBI nr database using blastx. Second, scallop sequences and the phototransduction/circadian clock gene sequences downloaded from NCBI (Table S1) were blasted against two available molluscan genomes (*C. gigas,* [45] and *L. gigantea,* http://genome.jgi-psf.org/Lotgi1/Lotgi1.download.html), then the top five hits from each blast were examined. Scallop sequences were considered homologous to the light detection pathway genes if the scallop sequences and the phototransduction/circadian clock genes shared a top-five hit in the molluscan genome BLAST results.

Orthologous gene sequences between the two scallop eye datasets, as well as between each scallop transcriptome and the predicted gene models from the *L. gigantea* genome, the CNS ESTs of *L. stagnalis* [44], and the *O. vulgaris* eye transcriptome [6], were found using the stand-alone program InParanoid v. 4.0 (http://inparanoid.sbc.su.se/cgi-bin/index.cgi; [49], [50], which was altered to use the tblastx BLAST algorithm. The analysis was run twice, first using a 50% minimum sequence overlap and then applying a 25% minimum sequence overlap to examine its effect on ortholog identification.

## Supporting Information

Figure S1 **Amino acid alignments of scallop Gq-protein coupled phototransduction genes to known homologs.** Phototransduction gene sequences from the *Placopecten magellanicus* adult eye transcriptome were translated and aligned to known homologs from *Drosophila*. Alignments were completed and exported from Geneious v. 5.6 (www.geneious.com). The graph above the alignment represents mean pairwise identity of each residue pair in each column, with green bars representing identical residues and missing bars highlighting areas where the sequences differ. End gaps in the alignments have solid green bars. Amino acids are shaded based on similarity, with black representing identical residues between the two sequences, grey representing similar residues, and white representing dissimilar residues. *A = arrestin, B = calmodulin, C = DAG kinase, D = Gq-protein alpha subunit, E = G-protein beta subunit, F = G-protein gamma subunit, G = opsin, H = phospholipase C, I = PI kinase, J = PI synthase, K = PI transferase, L = PIP kinase, M = protein kinase C, N = rhodopsin kinase, O = TRP, P = TRPL.)*
(PDF)

Figure S2 **Amino acid alignments of scallop Gi-protein coupled phototransduction genes to known homologs.** Phototransduction gene sequences from the *Placopecten magellanicus* adult eye transcriptome were translated and aligned to known homologs from *Drosophila*, human, or rat. Alignments were completed and exported from Geneious v. 5.6 (www.geneious.com). The graph above the alignment represents mean pairwise identity of each residue pair in each column, with green bars representing identical residues and missing bars highlighting areas where sequences differ. End gaps in the alignments have solid green bars. Amino acids are shaded based on similarity, with black representing identical residues between the two sequences, grey representing similar residues, and white representing dissimilar residues. *A = arrestin, B = cGMP gated channel alpha, C = cGMP gated channel beta, D = Go-protein alpha subunit, E = Gt-protein alpha subunit, F = G-protein beta subunit, G = G-protein gamma subunit, H = opsin, I = PDE alpha, J = PDE beta, K = protein kinase A, L = rhodopsin kinase.*
(PDF)

Figure S3 **Amino acid alignments of scallop circadian clock genes to known homologs.** Circadian clock gene sequences from the *Placopecten magellanicus* adult eye transcriptome were translated and aligned to known homologs from *Drosophila*, mouse, or *Crassostrea gigas*. Alignments were completed and exported from Geneious v. 5.6 (www.geneious.com). The graph above the alignment represents mean pairwise identity of each residue pair in each column, with green bars representing identical residues and missing bars highlighting areas where the sequences differ. End gaps in the alignments have solid green bars. Amino acids are shaded based on similarity, with black representing identical residues between the two sequences, grey representing similar residues, and white representing dissimilar residues. *A = clock, B = cryptochrome, C = cycle, D = doubletime, E = period, F = timeless.*
(PDF)

Table S1 **List of phototransduction and circadian clock protein sequences used in blasts to identify homologs within each scallop eye transcriptome.** All protein sequences were downloaded from NCBI and blasted against the transcriptomes using Geneious v. 5.5 (Biomatters).
(XLSX)

Table S2 **Genes annotated with Gene Ontology (GO) terms related to neural processes, vision, or retina(l) in the adult eye transctriptome of** ***A. irradians*** **.**
(XLSX)

Table S3 **Genes annotated with Gene Ontology (GO) terms related to neural processes, vision, or retina in the adult eye transcriptome of** ***P. magellanicus*** **.**
(XLSX)

Table S4 **List of genes identified in KEGG pathways related to light detection in the adult eye transcriptomes of** ***A. irradians*** **and** ***P. magellanicus*** **.** KEGG pathways were identified using the KEGG Automatic Annotation Server (www.genome.jp/tools/kaas).
(XLSX)
