# Supplementary material for: De Novo Assembly and Characterization of Two Transcriptomes Reveal Multiple Light-Mediated Functions in the Scallop Eye (Bivalvia: Pectinidae)
Source: PLoS One. 2013 Jul 29;8(7):e69852. doi: 10.1371/journal.pone.0069852 (PMC3726758; doi:10.1371/journal.pone.0069852)

Supplemental Figure 1. Amino acid alignments of scallop Gq-protein coupled phototransduction genes to known homologs. Phototransduction gene sequences from the *Placopecten magellanicus* adult eye transcriptome were translated and aligned to known homologs from *Drosophila*. Alignments were completed and exported from Geneious v. 5.6 (www.geneious.com). The graph above the alignment represents mean pairwise identity of each residue pair in each column, with green bars representing identical residues and missing bars highlighting areas where the sequence is different. End gaps in the alignments have solid green bars. Amino acids are shaded based on similarity, with black representing identical residues between the two sequences, grey representing similar residues, and white representing dissimilar residues. *A=arrestin, B=calmodulin, C=DAG kinase, D=Gq-protein alpha subunit, E=G-protein beta subunit, F=G-protein gamma subunit, G=opsin, H=phospholipase C, I=PI kinase, J=PI synthase, K=PI transferase, L=PIP kinase, M=protein kinase C, N=rhodopsin kinase, O=TRP, P=TRPL.*

A

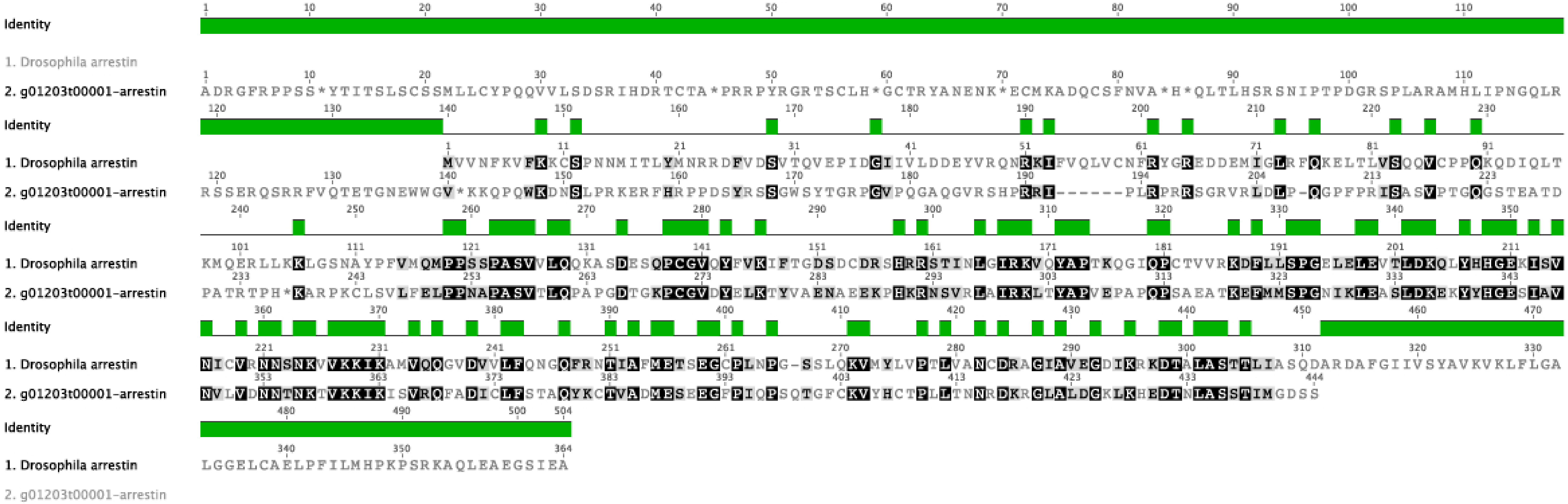

B

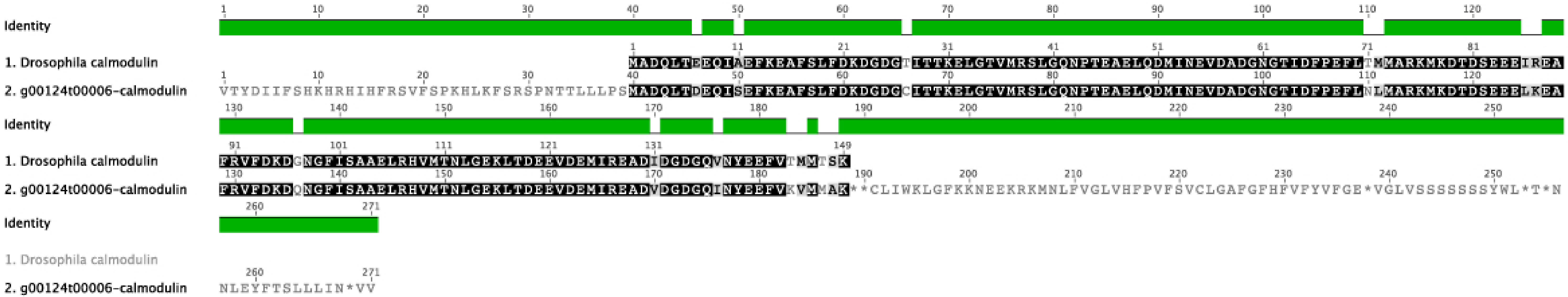
Identity
1. Drosophila calmodulin
MADQLTEEQIAEFKEAFSLFDKDGDGTITTKELGTVMRSLGQNPTEAELQDMINEVDADGNGTIDFPEFLTMMARKMKDTDSEEEIREA
2. g00124t00006-calmodulin
VTYDIIFSHKHRHIHFRSVFSPKHLKFSRSPNTTLLLPSMADQLTDEQISEFKEAFSLFDKDGDGCITTKELGTVMRSLGQNPTEAELQDMINEVDADGNGTIDFPEFLNLMARKMKDTDSEEELKEA
Identity
1. Drosophila calmodulin
FRVFDKDGNGFISAAELRHVMTNLGEKLTDEEVDEMIREADIDGDGQVNYEEFVTMMTSK
2. g00124t00006-calmodulin
FRVFDKDQNGFISAAELRHVMTNLGEKLTDEEVDEMIREADVDGDGQINYEEFVKVMMAK**CLIWKLGFKKNEEKRKMNLFVGLVHFPVFSVCLGAFGFHFVFYVFGE*VGLVSSSSSSSYWL*T*N
Identity
1. Drosophila calmodulin
2. g00124t00006-calmodulin
NLEYFTSLLLIN*VV

C

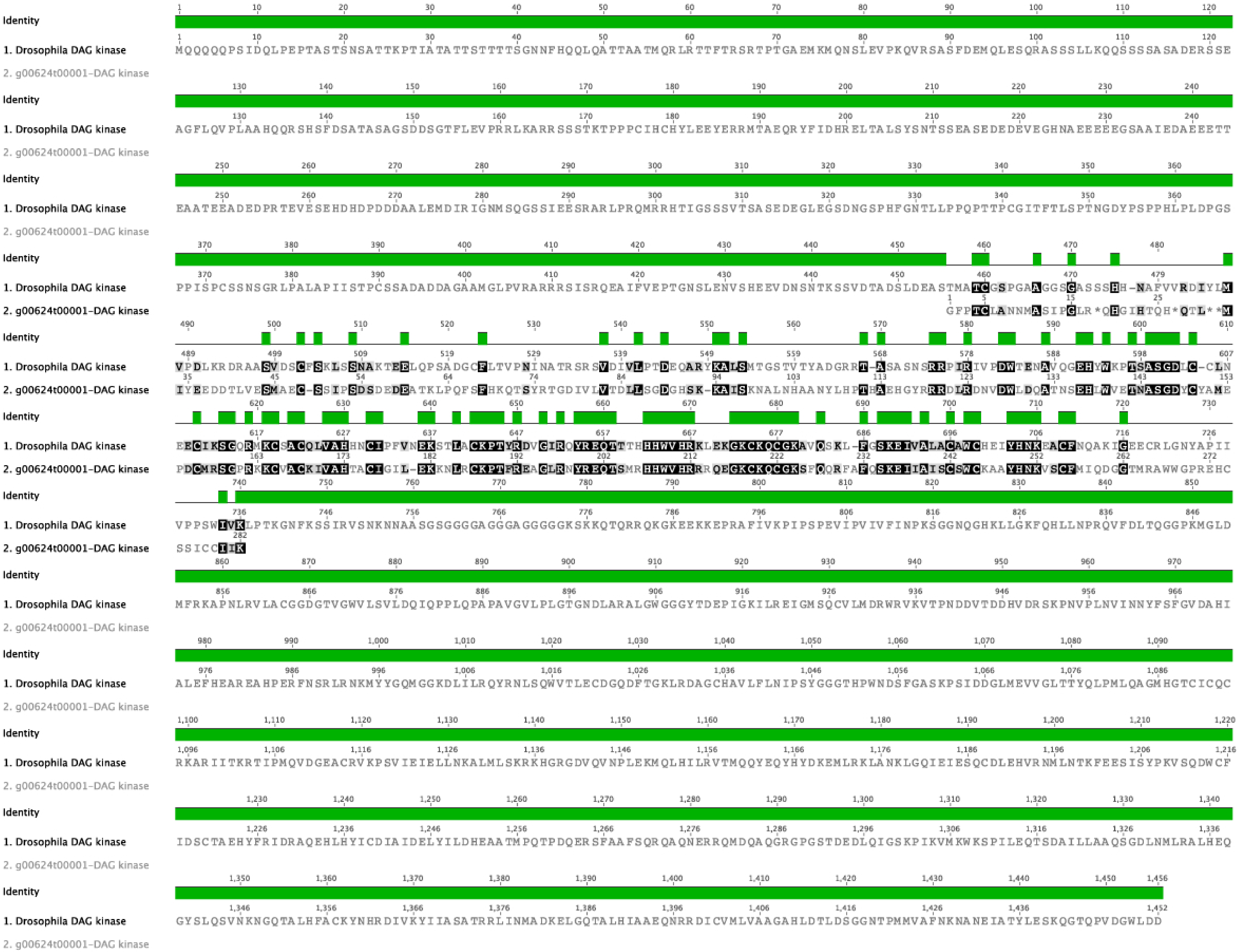
Identity
1. Drosophila DAG kinase
2. g00624t00001-DAG kinase

D

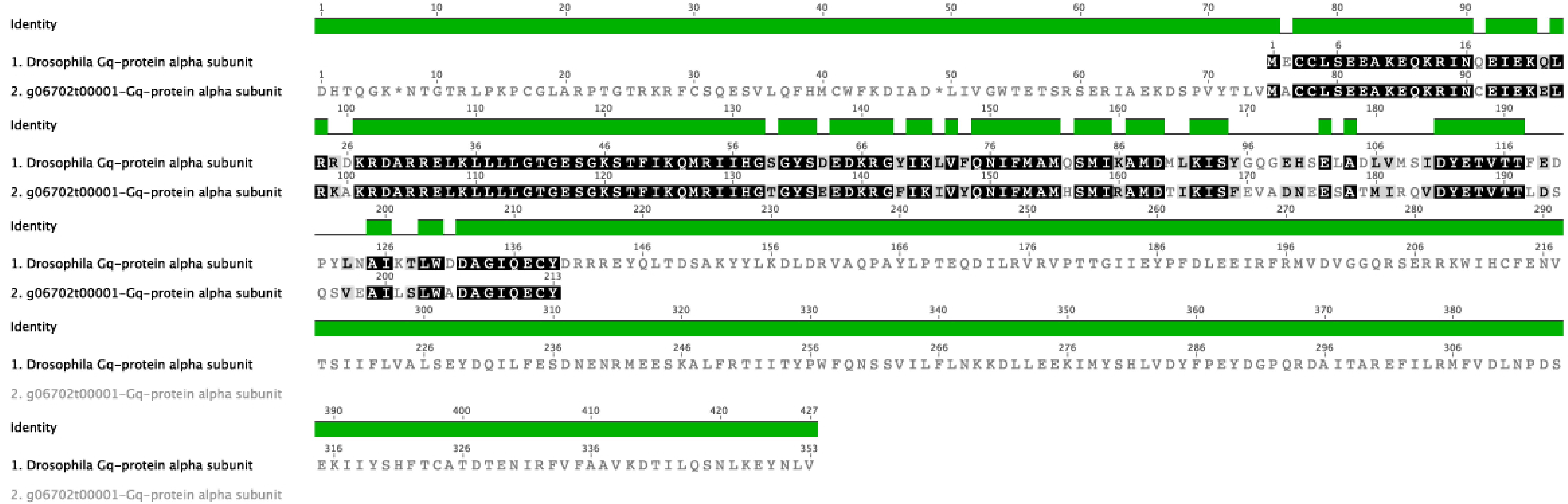

Identity
1. Drosophila Gq-protein alpha subunit
2. g06702t00001-Gq-protein alpha subunit

E

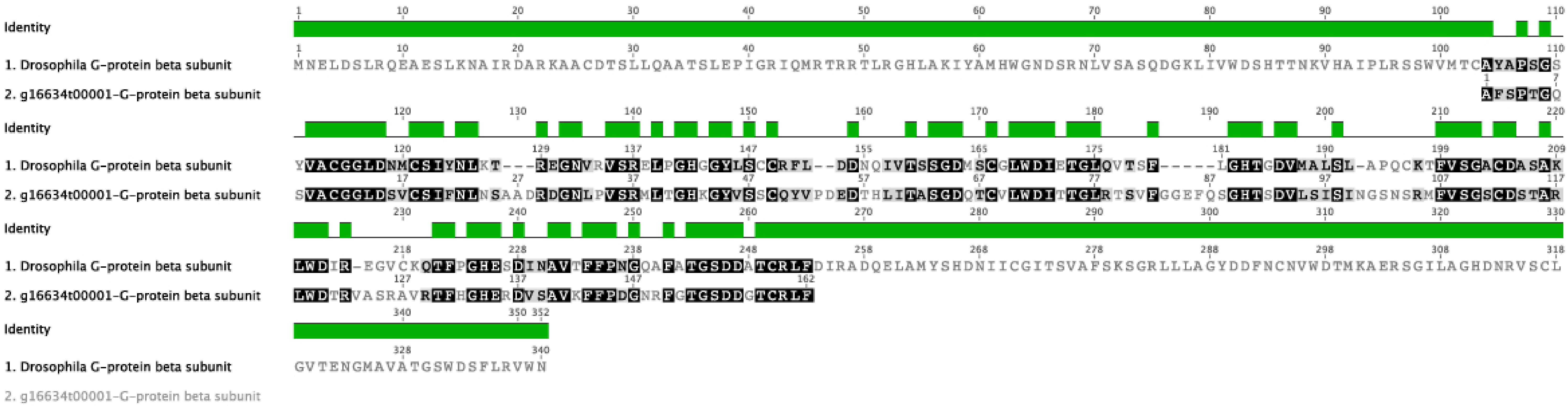

F

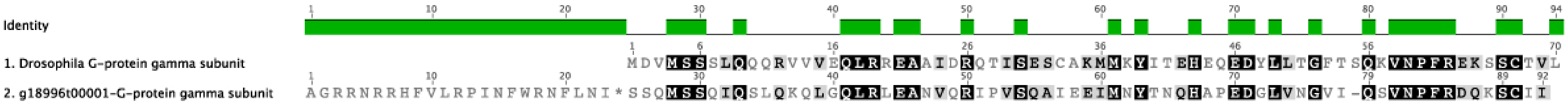
Identity
1. Drosophila G-protein gamma subunit
MDVMSSSLQQQRVVVEQLRREAAIDRQTISESCAKMMKYITEHEQEDYLLTGFTSQKVNPFREKSSCTVL
2. g18996t00001-G-protein gamma subunit
AGRRNRRHFVLRPINFWRNFLNI*SSQMSSQIQSLQKQLGQLRLEANVQRIPVSQAIEEIMNYTNQHAPEDGLVNGVI-QSVNPFRDQKSCII

G

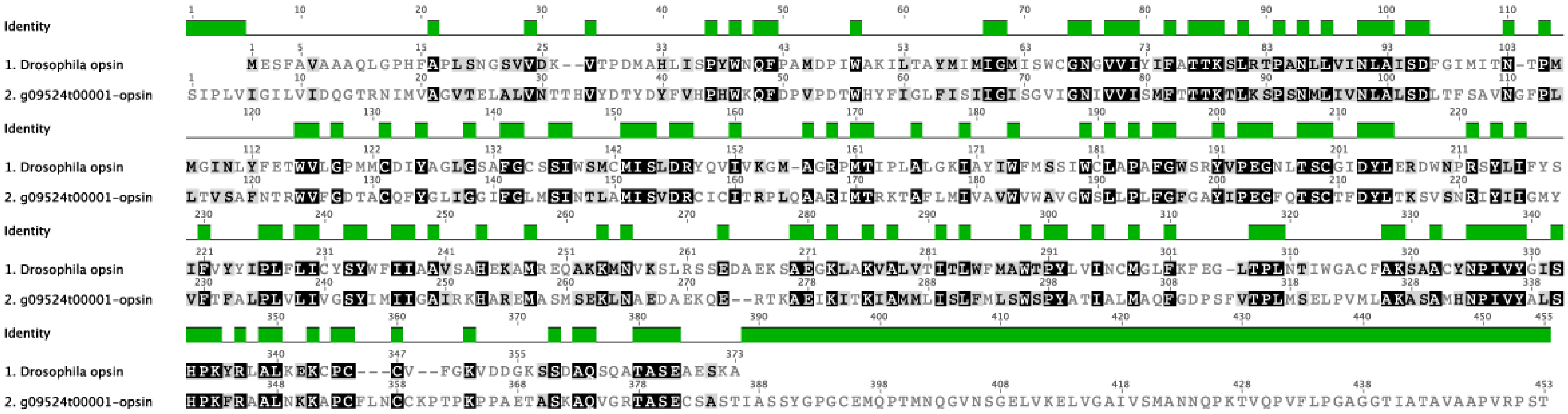

H

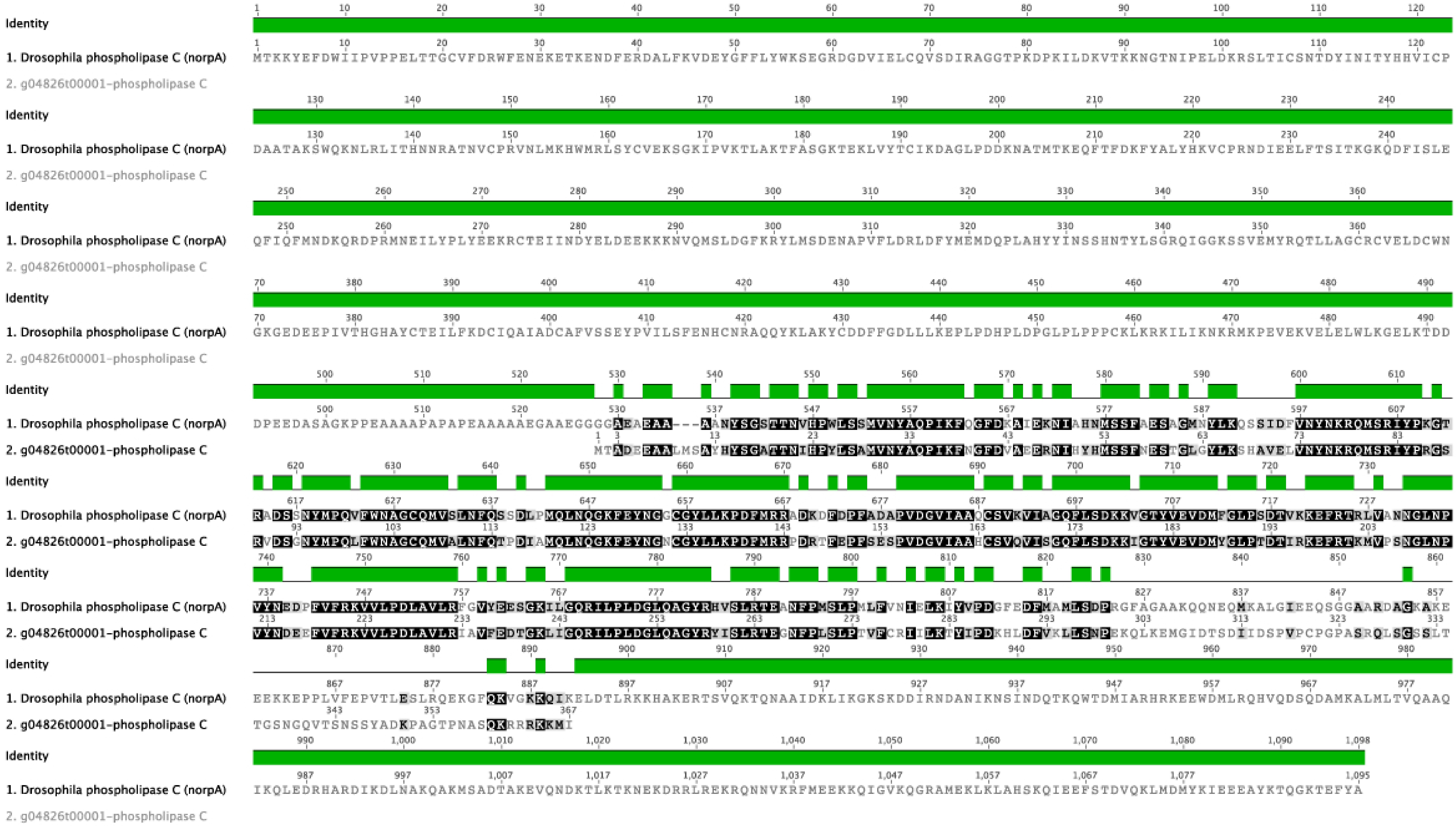
Identity
1. Drosophila phospholipase C (norpA)
2. g04826t00001-phospholipase C

I

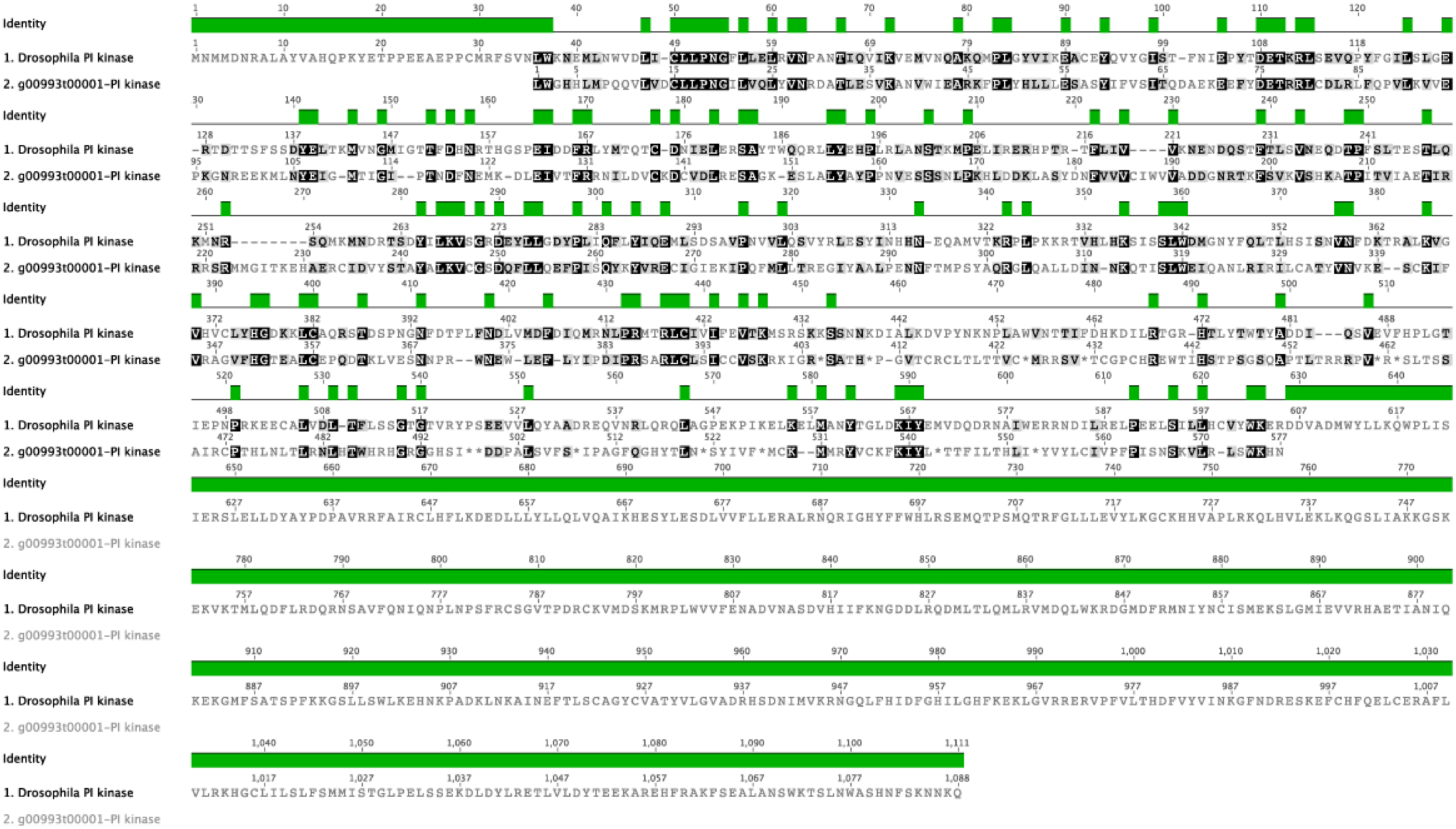

J

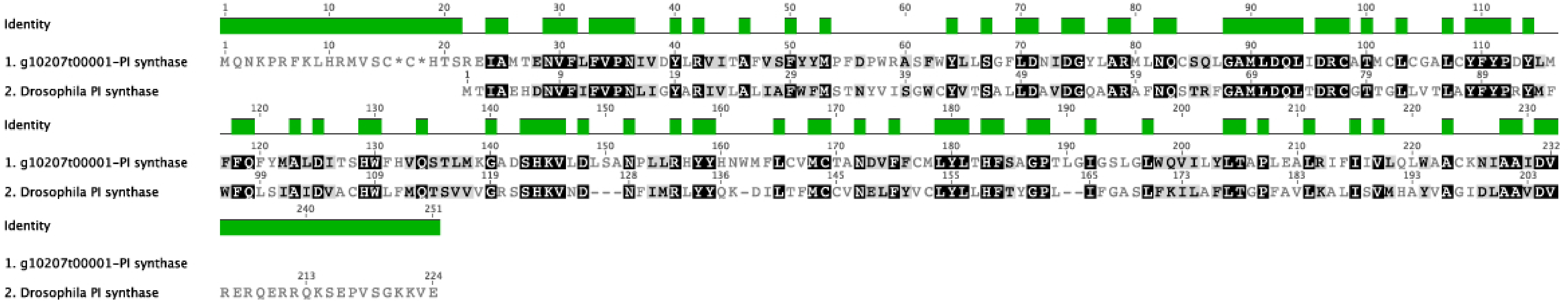
Identity
1. g10207t00001-PI synthase
MQNKPRFKLHRMVSC*C*HTSREIAMTENVFLFVPNIVDYLRVITAFVSFYYMPFDPWRASFWYLLSGFLDNIDGYLARMLNQCSQLGAMLDQLIDRCATMCLCGALCYFYPDYLM
2. Drosophila PI synthase
MTIAEHDNVFIFVPNLIGYARIVLALIAFWFMSTNYVISGWCYVTSALLDAVDGQAARAFNQSTRFGAMLDQLTDRCGTTGLLVTLAYFYPRYMF
Identity
1. g10207t00001-PI synthase
FFQFYMALDITSHWFHVQSTLMKGADSHKVLDLSANPLLRHYYHNWMFLCVMCTANDVFFCMLYLTHFSAGPTLGIGSLGLWQVILYLTAPLEALRIFIIVLQLWAACKNIAAIDV
2. Drosophila PI synthase
WFQLSIAIDVACHWLFMQTSVVVGRSSHKVND---NFIMRLYYQK-DILTFMCCVNELFYVCLYLLHFTYGPL--IFGASLFKILAFLTGPFAVLKALISVMHAYVAGIDLAAVDV
Identity
1. g10207t00001-PI synthase
2. Drosophila PI synthase
RERQERRQKSEPVSGKKVE

K

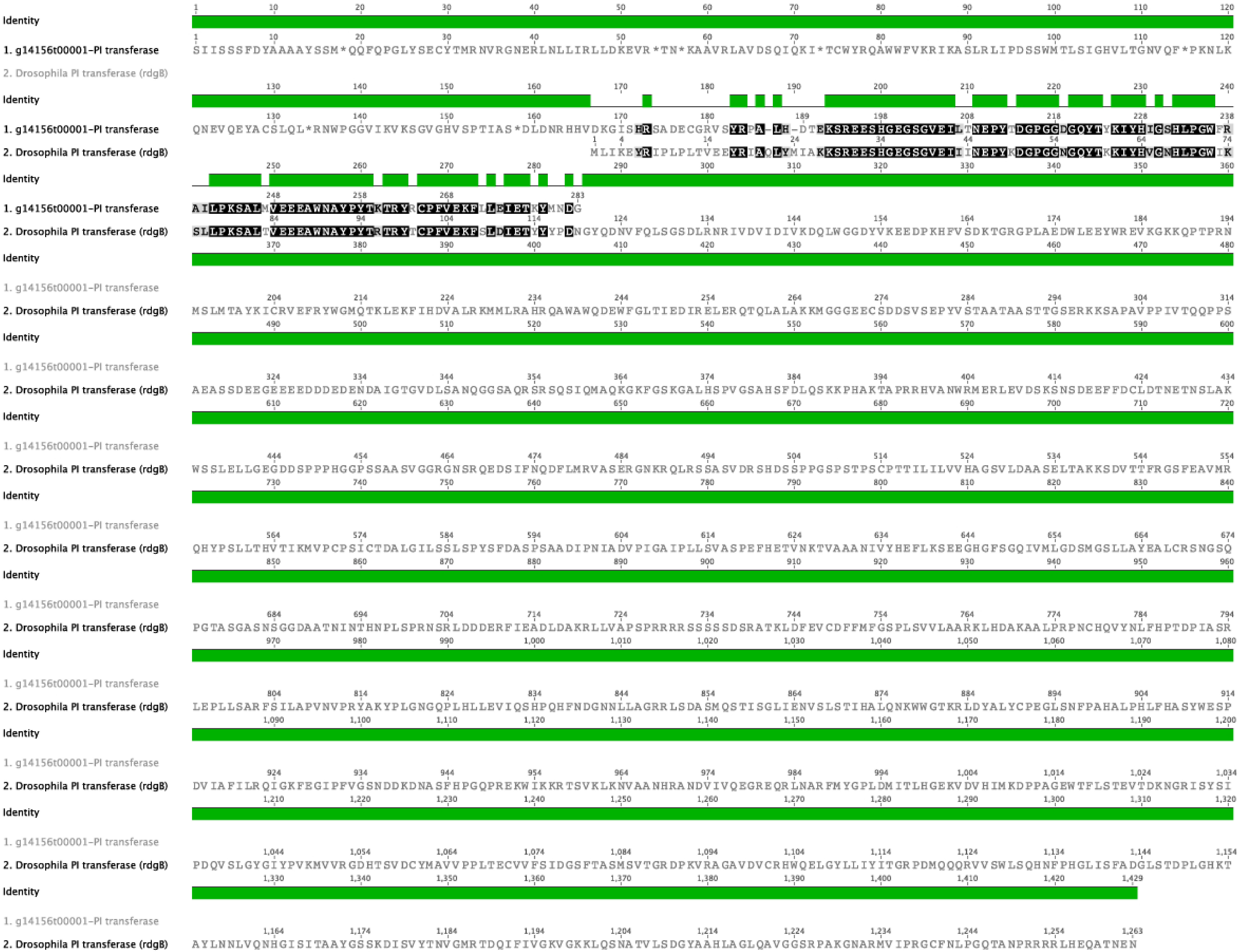
Identity
1. g14156t00001–PI transferase
2. Drosophila PI transferase (rdgB)

L

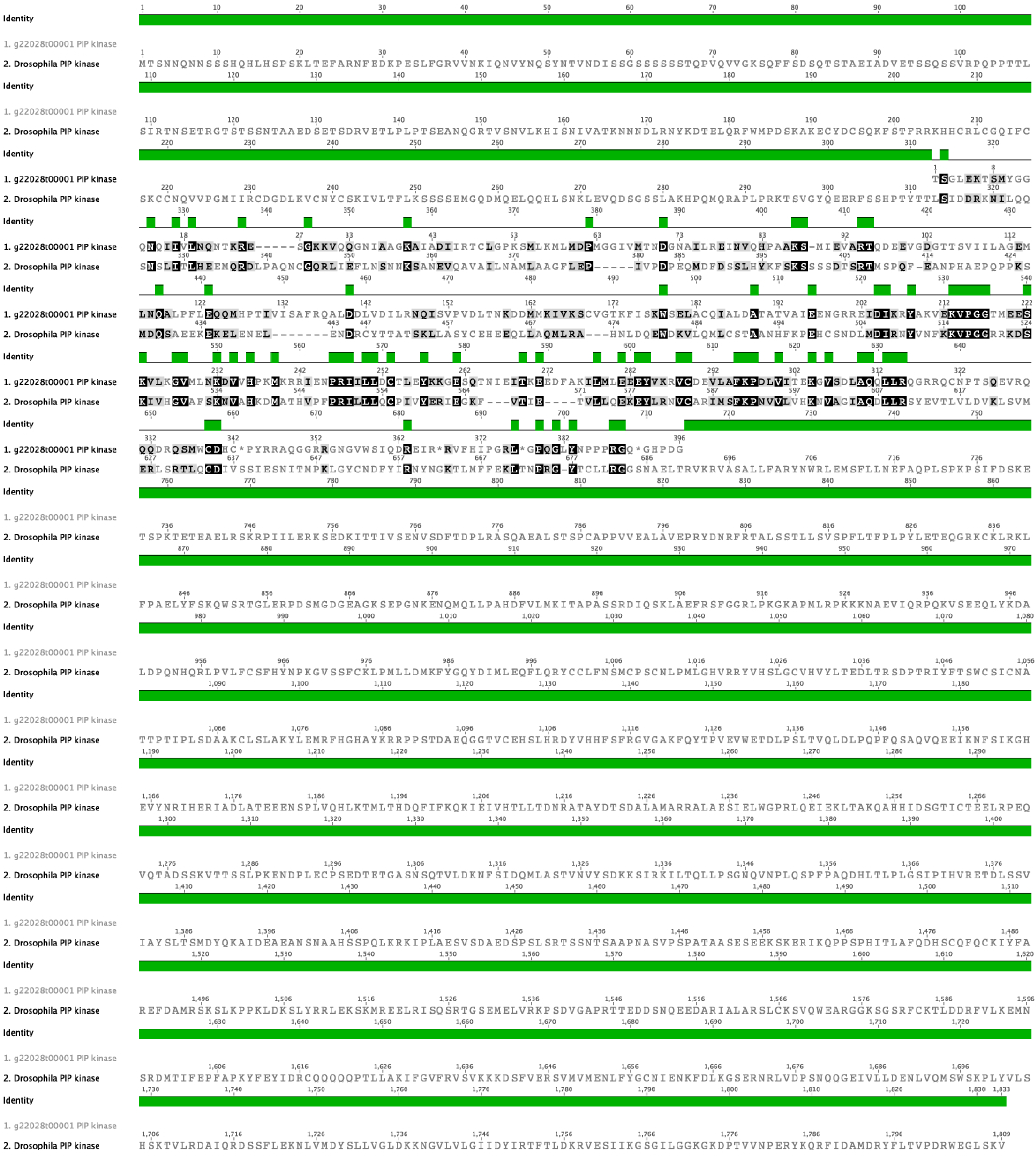
Identity
1. g22028t00001 PIP kinase
2. Drosophila PIP kinase

M

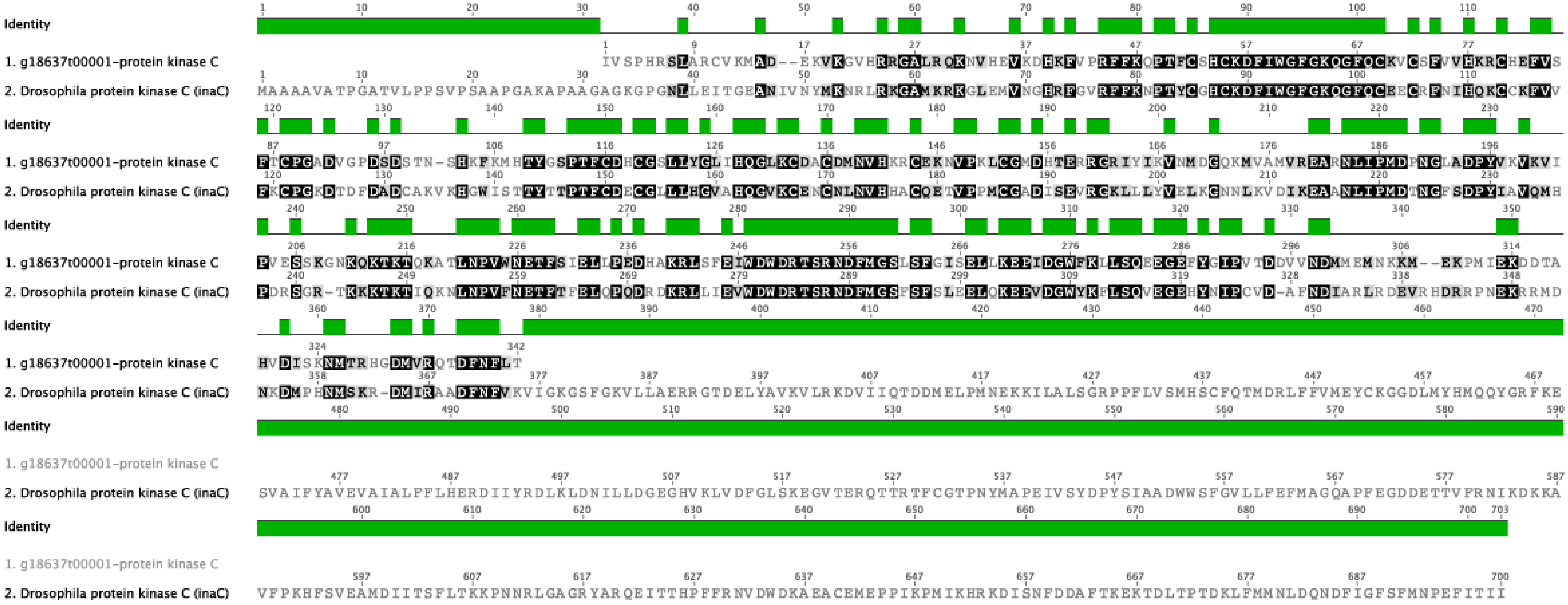
Identity
1. g18637t00001-protein kinase C
2. Drosophila protein kinase C (inaC)

N

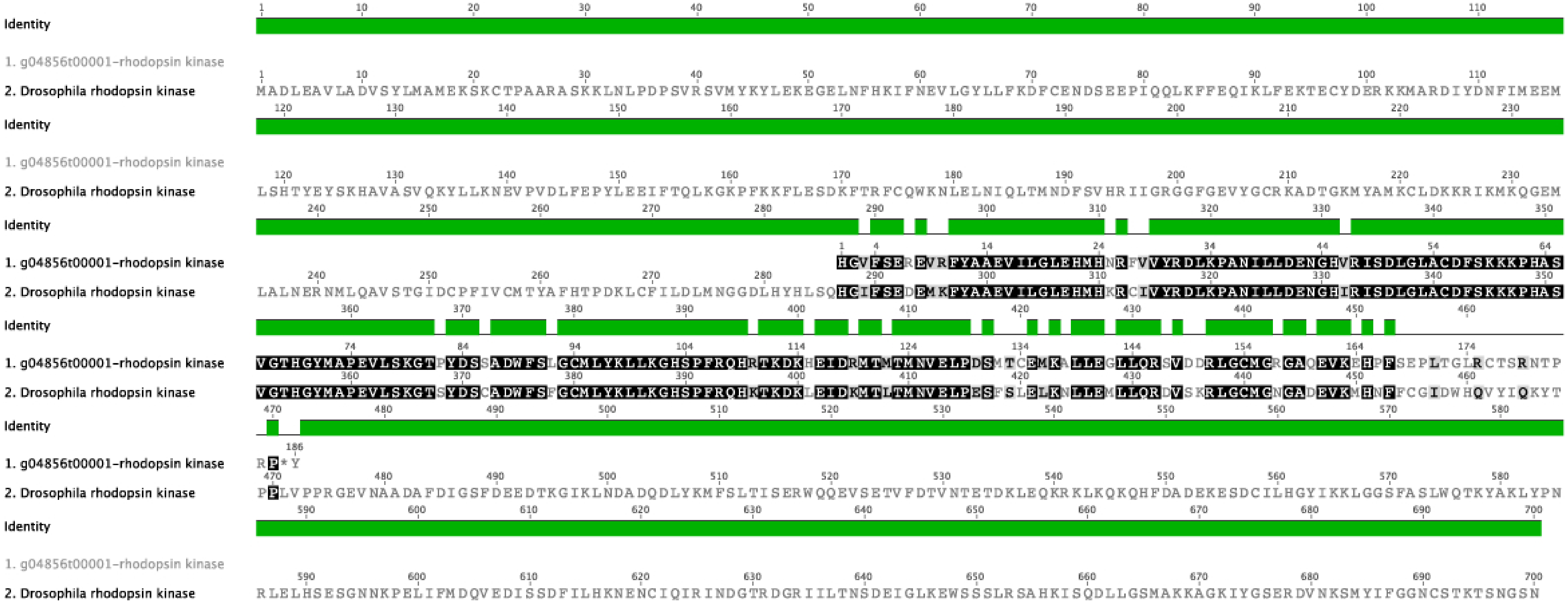

Identity
1. g04856t00001-rhodopsin kinase
2. Drosophila rhodopsin kinase

O

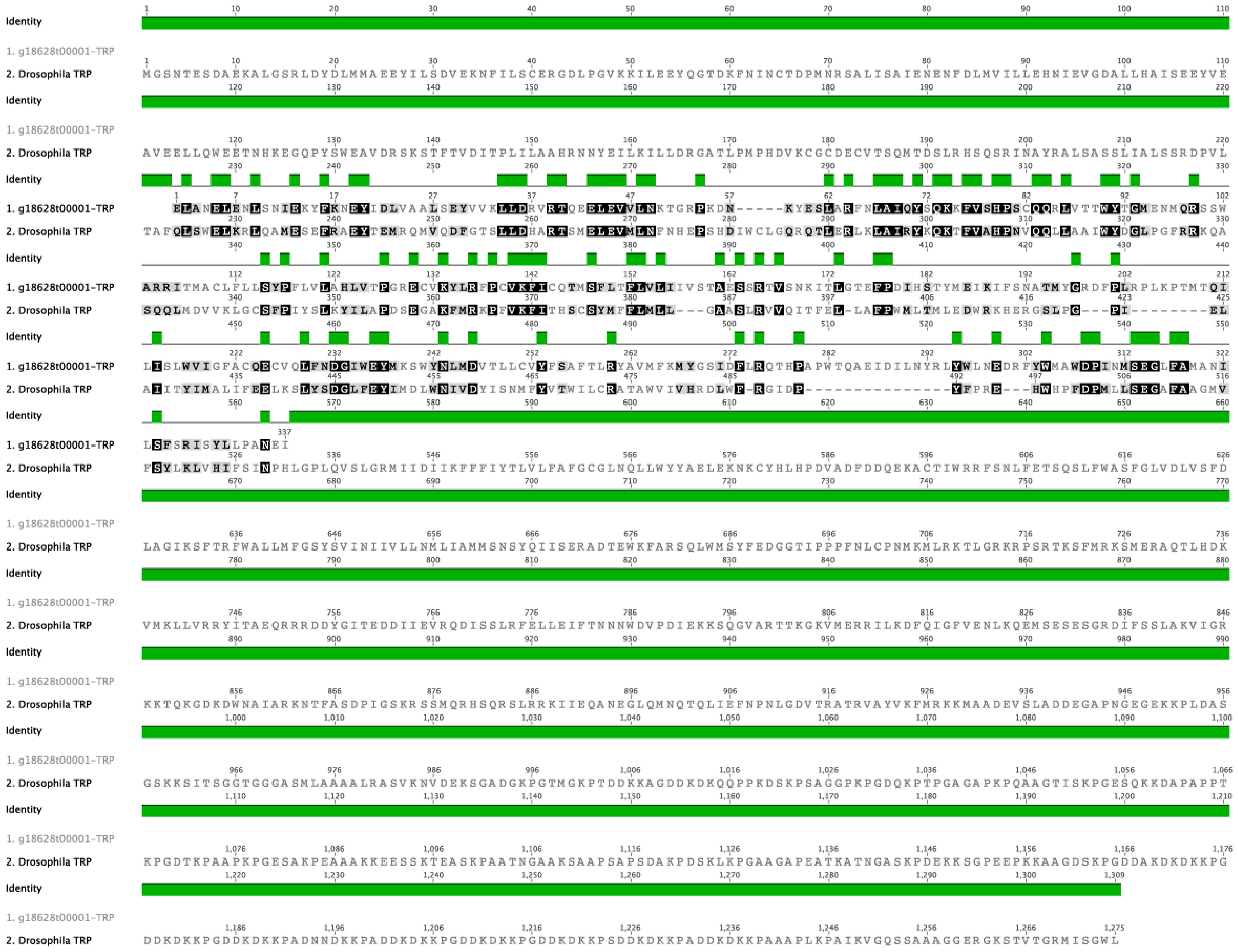

P

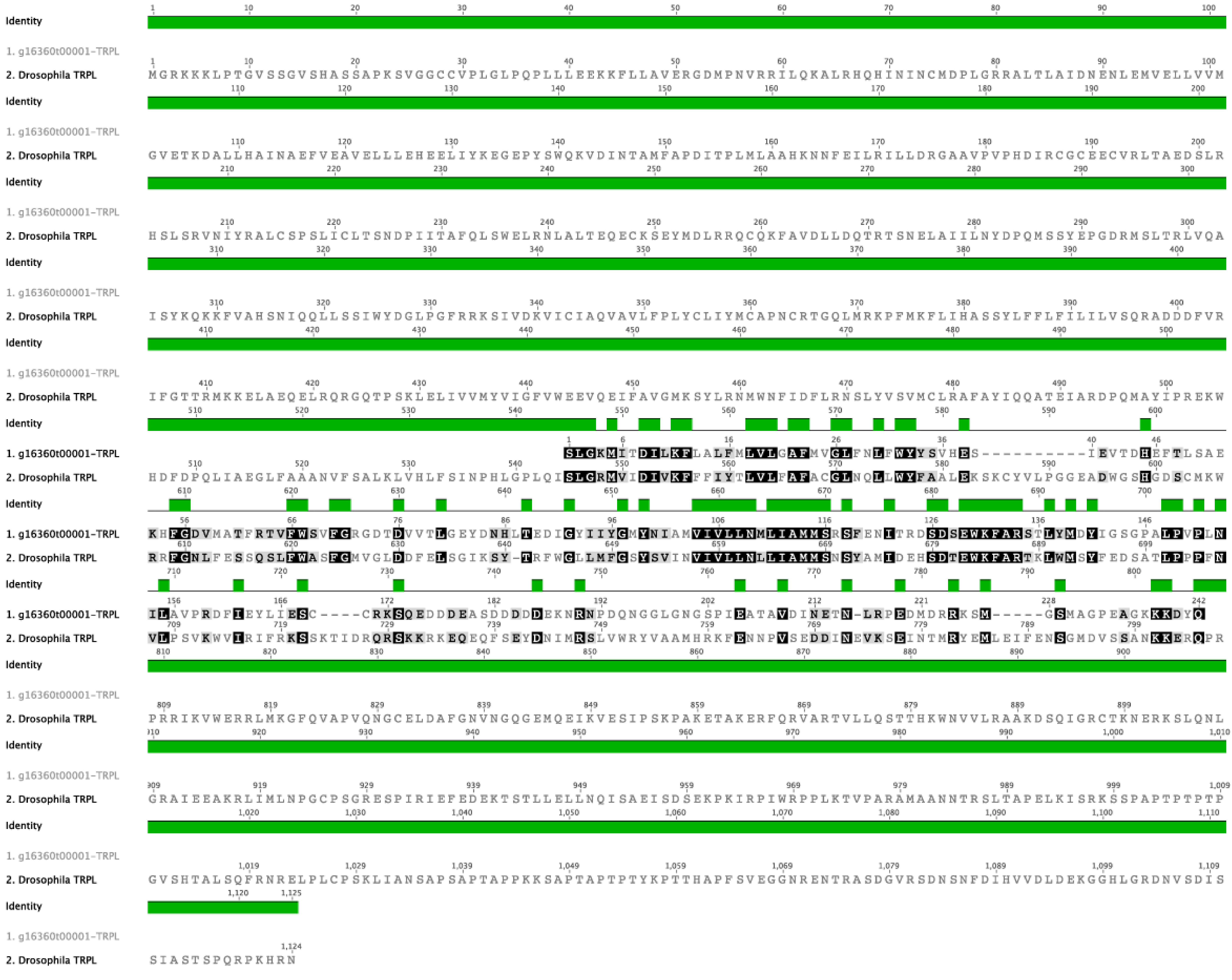

Supplement: Figure S1 — Amino acid alignments of scallop Gq-protein coupled phototransduction genes to known homologs. Phototransduction gene sequences from the Placopecten magellanicus adult eye transcriptome were translated and aligned to known homologs from Drosophila. Alignments were completed and exported from Geneious v. 5.6 (www.geneious.com). The graph above the alignment represents mean pairwise identity of each residue pair in each column, with green bars representing identical residues and missing bars highlighting areas where the sequences differ. End gaps in the alignments have solid green bars. Amino acids are shaded based on similarity, with black representing identical residues between the two sequences, grey representing similar residues, and white representing dissimilar residues. A = arrestin, B = calmodulin, C = DAG kinase, D = Gq-protein alpha subunit, E = G-protein beta subunit, F = G-protein gamma subunit, G = opsin, H = phospholipase C, I = PI kinase, J = PI synthase, K = PI transferase, L = PIP kinase, M = protein kinase C, N = rhodopsin kinase, O = TRP, P = TRPL.) (PDF) [file pone.0069852.s001.pdf]
