# Supplementary material for: De Novo Assembly and Characterization of Two Transcriptomes Reveal Multiple Light-Mediated Functions in the Scallop Eye (Bivalvia: Pectinidae)
Source: PLoS One. 2013 Jul 29;8(7):e69852. doi: 10.1371/journal.pone.0069852 (PMC3726758; doi:10.1371/journal.pone.0069852)
Supplement: Figure S2 — Amino acid alignments of scallop Gi-protein coupled phototransduction genes to known homologs. Phototransduction gene sequences from the Placopecten magellanicus adult eye transcriptome were translated and aligned to known homologs from Drosophila, human, or rat. Alignments were completed and exported from Geneious v. 5.6 (www.geneious.com). The graph above the alignment represents mean pairwise identity of each residue pair in each column, with green bars representing identical residues and missing bars highlighting areas where sequences differ. End gaps in the alignments have solid green bars. Amino acids are shaded based on similarity, with black representing identical residues between the two sequences, grey representing similar residues, and white representing dissimilar residues. A = arrestin, B = cGMP gated channel alpha, C = cGMP gated channel beta, D = Go-protein alpha subunit, E = Gt-protein alpha subunit, F = G-protein beta subunit, G = G-protein gamma subunit, H = opsin, I = PDE alpha, J = PDE beta, K = protein kinase A, L = rhodopsin kinase. (PDF) [file pone.0069852.s002.pdf]

Supplemental Figure 2. Amino acid alignments of scallop Gi-protein coupled phototransduction genes to known homologs. Phototransduction gene sequences from the *Placopecten magellanicus* adult eye transcriptome were translated and aligned to known homologs from *Drosophila*, human, or rat. Alignments were completed and exported from Geneious v. 5.6 (www.geneious.com). The graph above the alignment represents mean pairwise identity of each residue pair in each column, with green bars representing identical residues and missing bars highlighting areas where the sequence is different. End gaps in the alignments have solid green bars. Amino acids are shaded based on similarity, with black representing identical residues between the two sequences, grey representing similar residues, and white representing dissimilar residues. *A=arrestin, B=cGMP gated channel alpha, C=cGMP gated channel beta, D=Go-protein alpha subunit, E=Gt-protein alpha subunit, F=G-protein beta subunit, G=G-protein gamma subunit, H=opsin, I=PDE alpha, J=PDE beta, K=protein kinase A, L=rhodopsin kinase.*

A

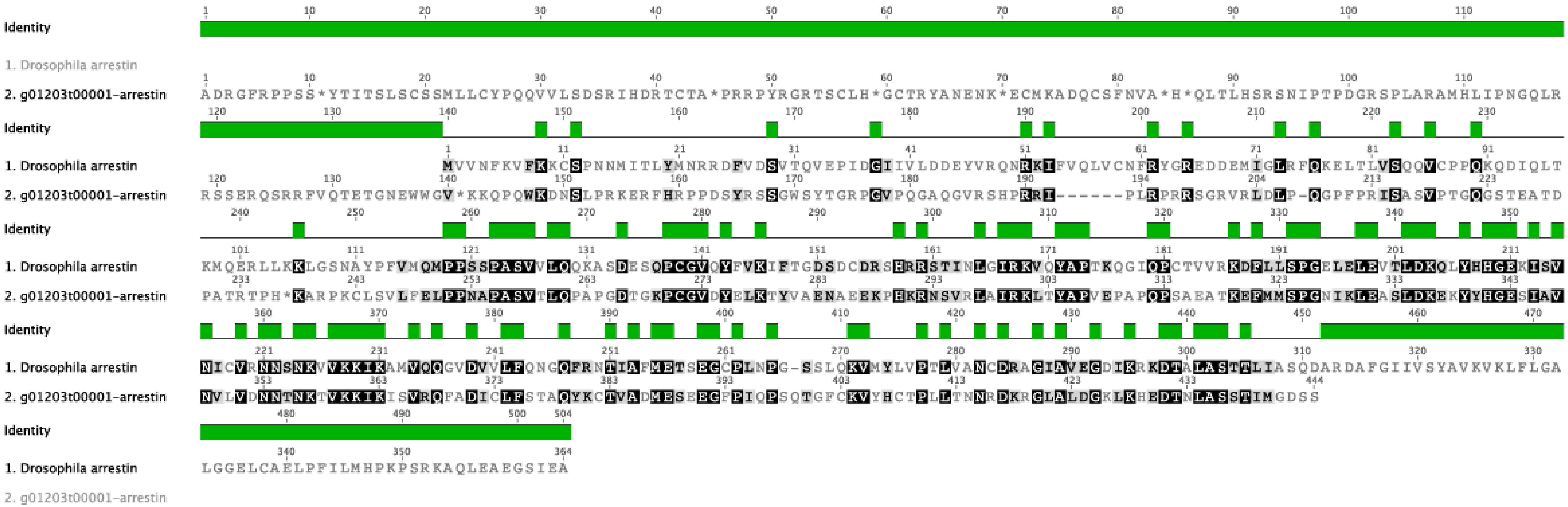

B

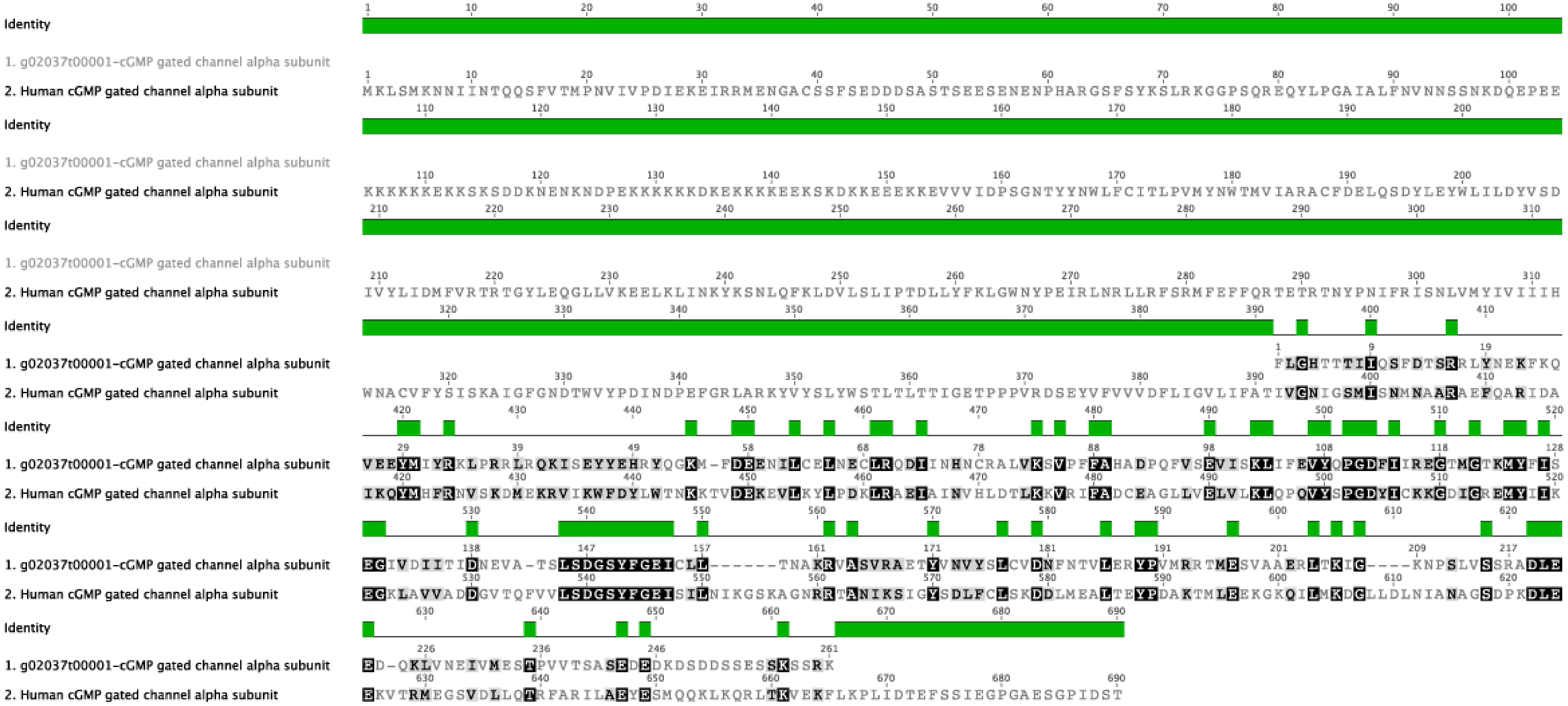
Identity
1. g02037t00001-cGMP gated channel alpha subunit
2. Human cGMP gated channel alpha subunit

C

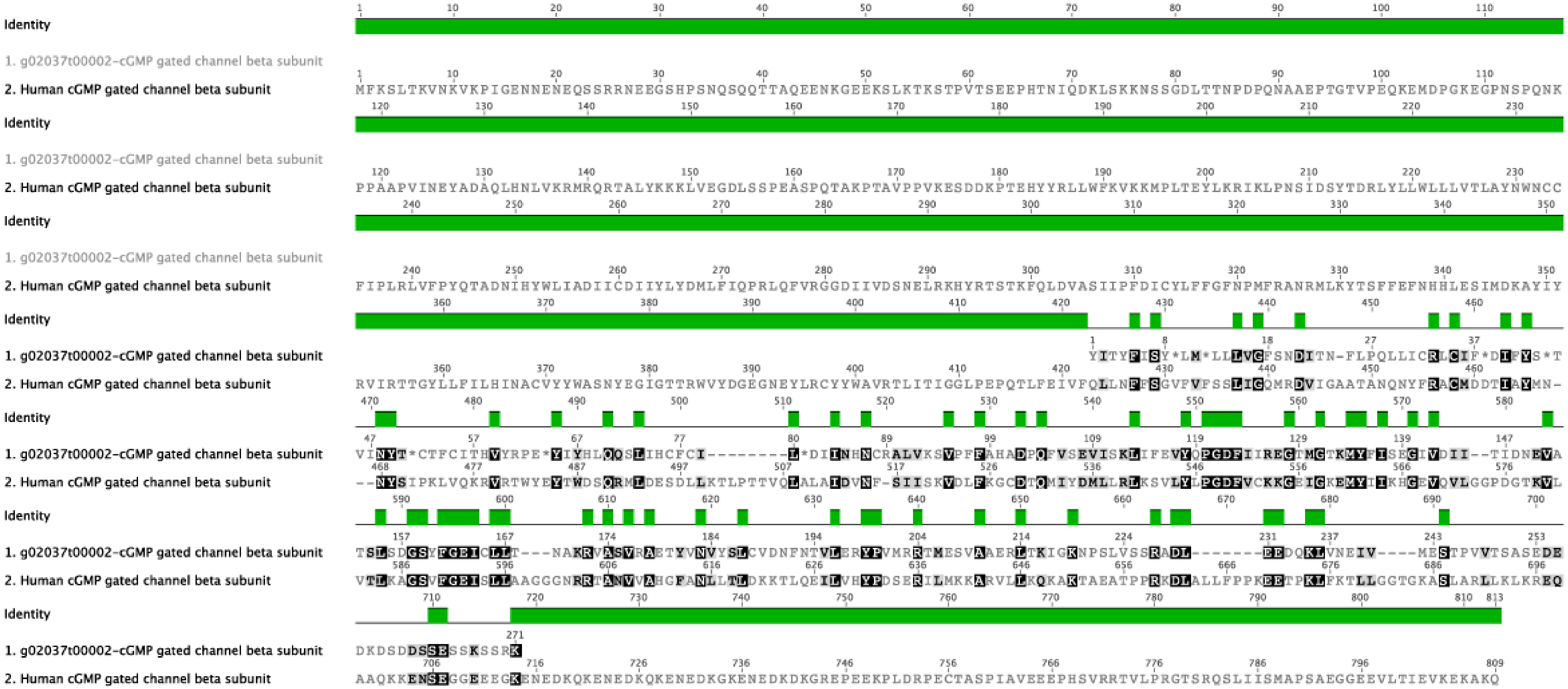

Identity
1. g02037t00002-cGMP gated channel beta subunit
2. Human cGMP gated channel beta subunit

D

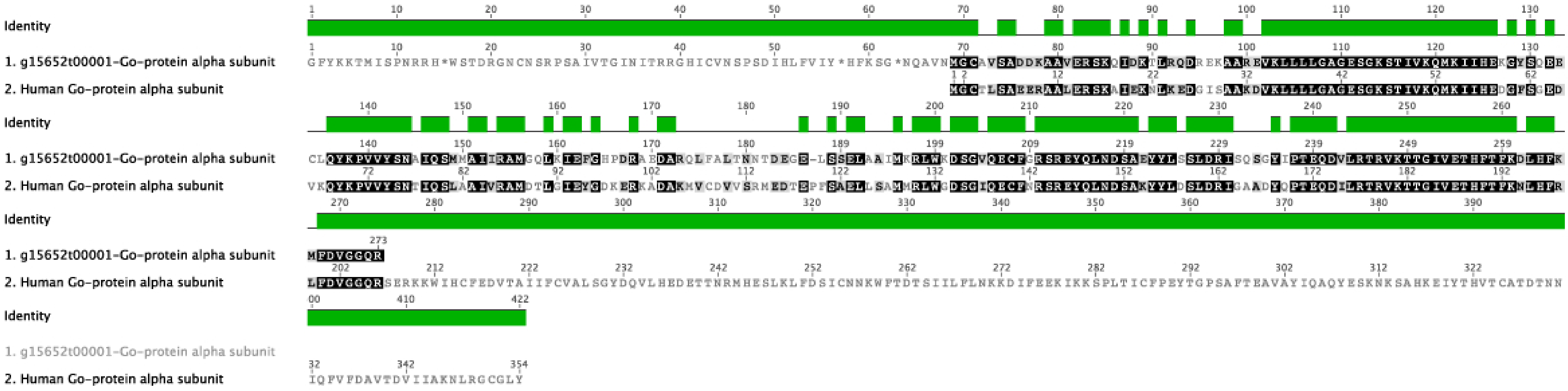
Identity
1. g15652t00001-Go-protein alpha subunit
2. Human Go-protein alpha subunit

E

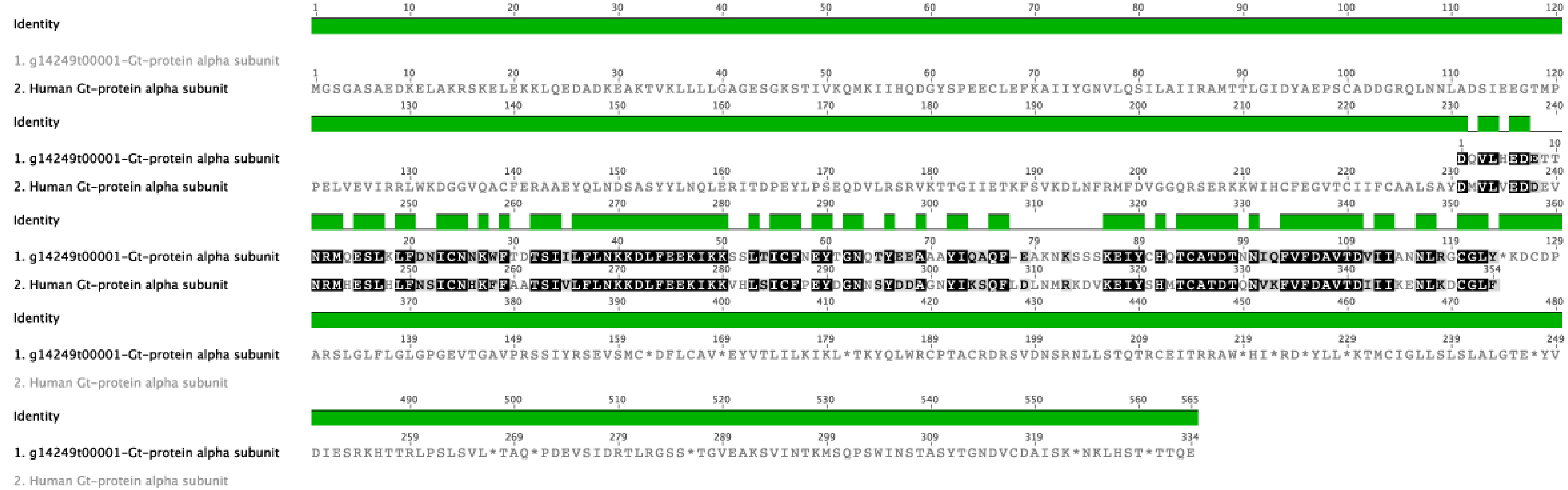
Identity
1. g14249t00001-Gt-protein alpha subunit
2. Human Gt-protein alpha subunit
MGSGASAEDKELAKRSKELEKKLQEDADKEAKTVKLLLLGAGESGKSTIVKQMKIIHQDGYSPEECLEFKAIIYGNVLQSILAIIRAMTTLGIDYAEPSCADDGRQLNNLADSIEEGTMP
Identity
1. g14249t00001-Gt-protein alpha subunit
DQVLHEDETT
2. Human Gt-protein alpha subunit
PELVEVIRRLWKDGGVQACFERAAEYQLNDSASYYLNQLERITDPEYLPSEQDVLRSRVKTTGIIETKFSVKDLNFRMFDVGGQRSERKKWIHCFEGVTCIIFCAALSAYDMVLVEDDEV
Identity
1. g14249t00001-Gt-protein alpha subunit
NRMQESLKLFDNICNNKWFTDTSIILFLNKKDLFEEKIKKSSLTICFNEYTGNQTYEEAAAYIQAQF-EAKNKSSSKEIYCHQTCATDTNNIQFVFDAVTDVIIANNLRGCGLY*KDCDP
2. Human Gt-protein alpha subunit
NRMHESLHLFNSICNHKFFAATSIVLFLNKKDLFEEKIKKVHLSICFPEYDGNNSYDDAGNYIKSQFLDLNMRKDVKEIYSHMTCATDTQNVKFVFDAVTDIIIKENLKDCGLF
Identity
1. g14249t00001-Gt-protein alpha subunit
ARSLGLFLGLGPGEVTGAVPRSSIYRSEVSMC*DFLCAV*EYVTLILKIKL*TKYQLWRCPTACRDRSVDNSRNLLSTQTRCEITRRAW*HI*RD*YLL*KTMCIGLLSLSLALGTE*YV
2. Human Gt-protein alpha subunit
Identity
1. g14249t00001-Gt-protein alpha subunit
DIESRKHTTRLPSLSVL*TAQ*PDEVSIDRTLRGSS*TGVEAKSVINTKMSQPSWINSTASYTGNDVCDAISK*NKLHST*TTQE
2. Human Gt-protein alpha subunit

F

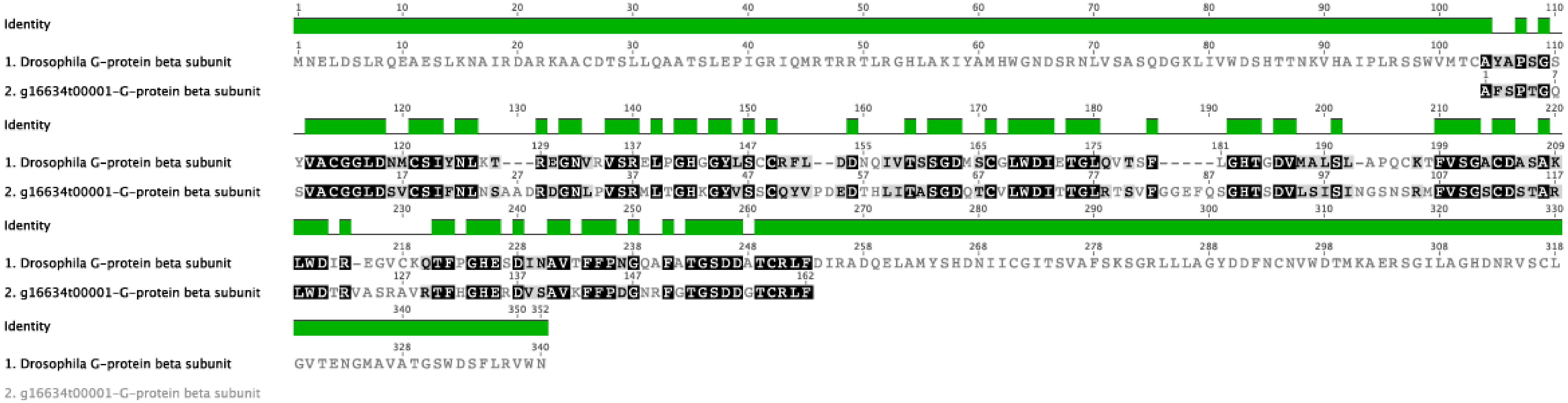

Identity
1. Drosophila G-protein beta subunit
MNELDSLRQEAESLKNAIRDARKAACDTSLLQAATSLEPIGRIQMRTRRTLRGHLAKIYAMHWGNDSRNLVSASQDGKLIVWDSHTTNKVHAIPLRSSWVMTCAYAPSGS
2. g16634t00001-G-protein beta subunit
AFSPTGQ
Identity
1. Drosophila G-protein beta subunit
YVACGGLDNMCSIYNLKT---REGNVRVSRELPGHGGYLSCCRFL--DDNQIVTSSGDMSCGLWDIETGLQVTSF-----LGHTGDVMALSL-APQCKTFVSGACDASAK
2. g16634t00001-G-protein beta subunit
SVACGGLDSVCSIFNLNSAADRDGNLPVSRMLTGHKGYVSSCQYVPDEDTHLITASGDQTCVLWDITTGLRTSVFGGEFQSGHTSDVLSISINGSNSRMFVSGSCDSTAR
Identity
1. Drosophila G-protein beta subunit
LWDIR-EGVCKQTFPGHESDINAVTFFPNGQAFATGSDDATCRLFDIRADQELAMYSHDNIICGITSVAFSKSGRLLLAGYDDFNCNVWDTMKAERSGILAGHDNRVSCL
2. g16634t00001-G-protein beta subunit
LWDTRVASRAVRTFHGHERDVSAVKFFPDGNRFGTGSDDGTCRLF
Identity
1. Drosophila G-protein beta subunit
GVTENGMAVATGSWDSFLRVWN
2. g16634t00001-G-protein beta subunit

G

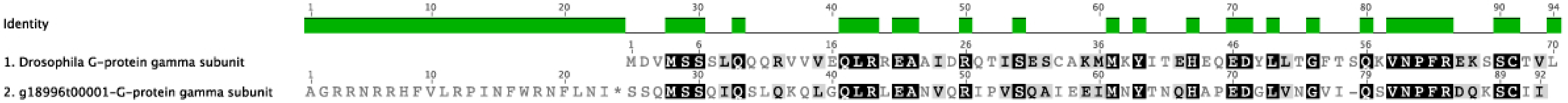

Identity
1. Drosophila G-protein gamma subunit
MDVMSSSLQQQRVVVEQLRREAAIDRQTISESCAKMMKYITEHEQEDYLLTGFTSQKVNPFREKSSCTVL
2. g18996t00001-G-protein gamma subunit
AGRRNRRHFVLRPINFWRNFLNI*SSQMSSQIQSLQKQLGQLRLEANVQRIPVSQAIEEIMNYTNQHAPEDGLVNGVI-QSVNPFRDQKSCII

H

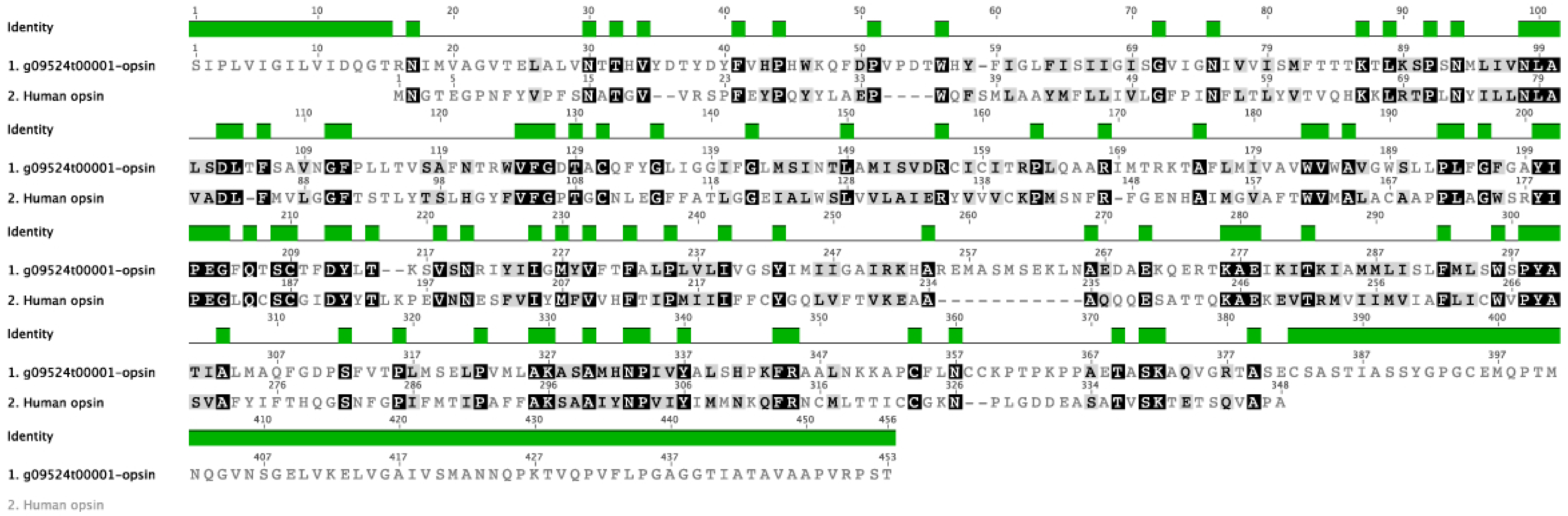

Identity
1. g09524t00001-opsin
SIPLVIGILVIDQGTRNIMVAGVTELALVNTTHVYDTYDYFVHPHWKQFDPVPDTWHY-FIGLFISIIGISGVIGNIVVISMFTTTKTLKSPSNMLIVNLA
2. Human opsin
MNGTEGPNFYVPFSNATGV--VRSPFEYPQYYLAEP----WQFSMLAAYMFLLIVLGFPINFLTLYVTVQHKKLRTPLNYILLNLA
1. g09524t00001-opsin
LSDLTFSAVNGFPLLTVSAFNTRWVFGDTACQFYGLIGGIFGLMSINTLAMISVDRCICITRPLQAARIMTRKTAFLMIVAVWVWAVGWSLLPLFGFGAYI
2. Human opsin
VADL-FMVLGGFTSTLYTSLHGYFVFGPTGCNLEGFFATLGGEIALWSLVVLAIERYVVVCKPMSNFR-FGENHAIMGVAFTWVMALACAAPPLAGWSRYI
1. g09524t00001-opsin
PEGFQTSCTFDYLT--KSVSNRIYIIGMYVFTFALPLVLIVGSYIMIIGAIRKHAREMASMSEKLNAEDAEKQERTKAEIKITKIAMMLISLFMLSWSPYA
2. Human opsin
PEGLQCSCGIDYYTLKPEVNNESFVIYMFVVHFTIPMIIIFFCYGQLVFTVKEAA-----------AQQQESATTQKAEKEVTRMVIIMVIAFLICWVPYA
1. g09524t00001-opsin
TIALMAQFGDPSFVTPLMSELPVMLAKASAMHNPIVYALSHPKFRAALNKKAPCFLNCCKPTPKPPAETASKAQVGRTASECSASTIASSYGPGCEMQPTM
2. Human opsin
SVAFYIFTHQGSNFGPIFMTIPAFFAKSAAIYNPVIYIMMNKQFRNCMLTTICCGKN--PLGDDEASATVSKTETSQVAPA
1. g09524t00001-opsin
NQGVNSGELVKELVGAIVSMANNQPKTVQPVFLPGAGGTIATAVAAPVRPST
2. Human opsin

I

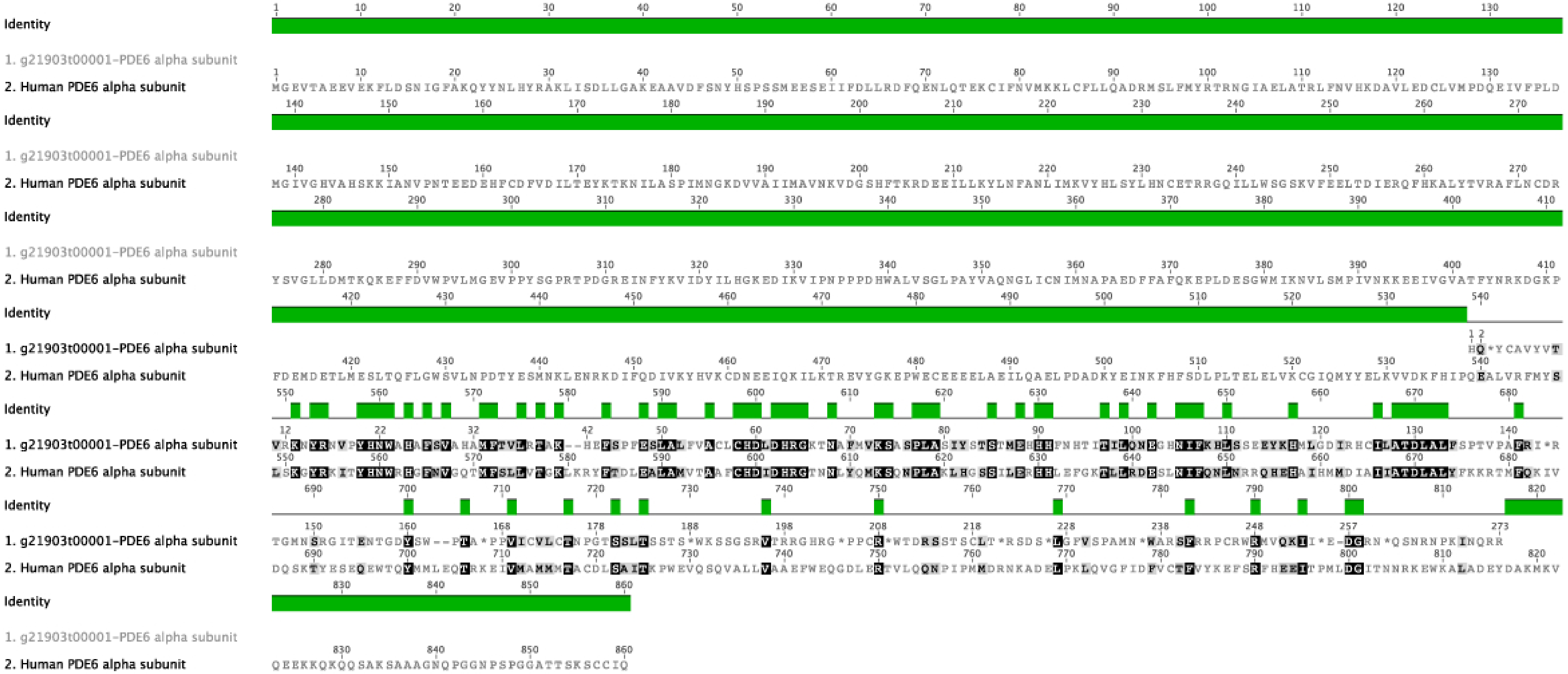

Identity
1. g21903t00001-PDE6 alpha subunit
2. Human PDE6 alpha subunit

J

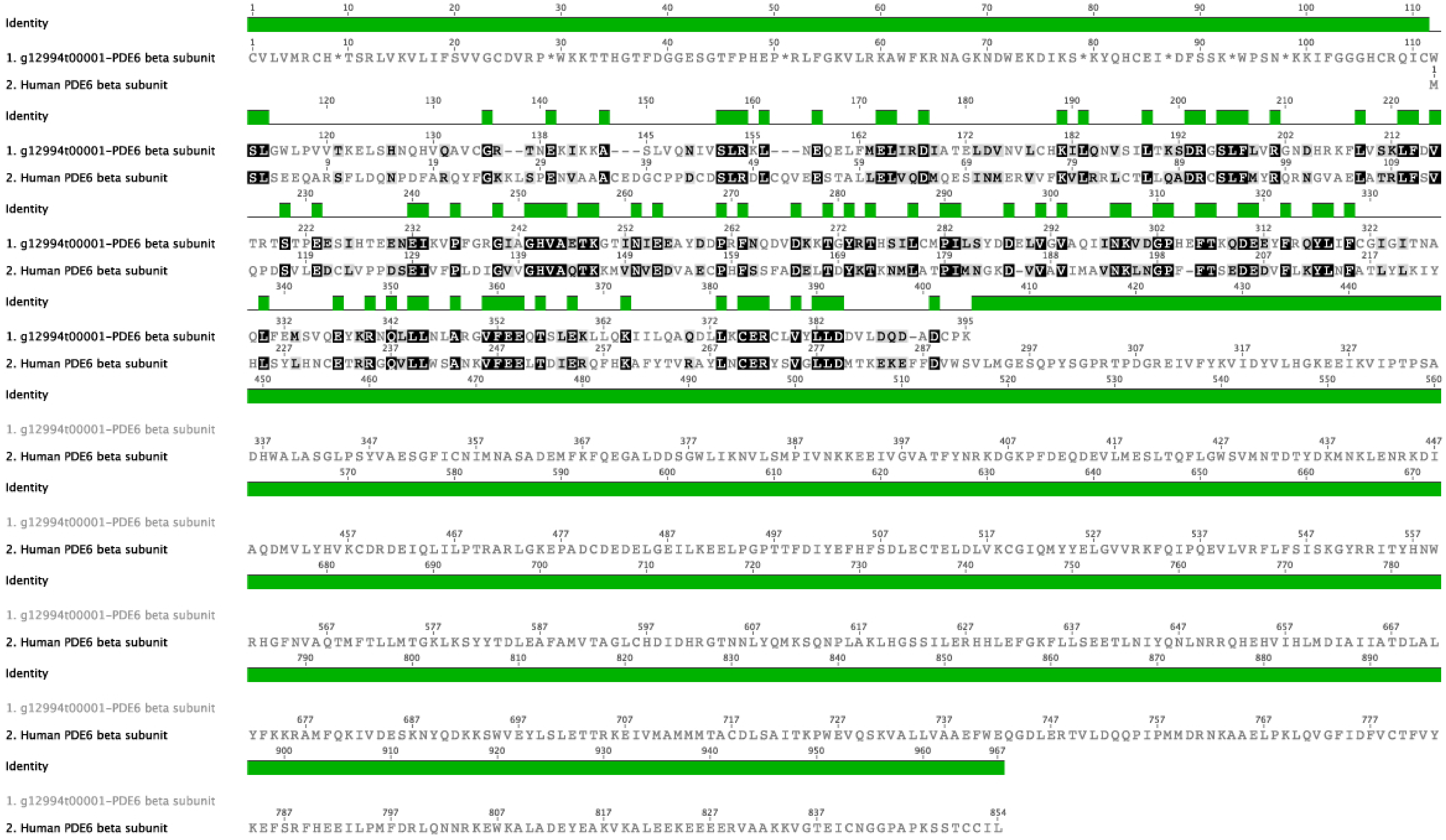
Identity
1. g12994t00001-PDE6 beta subunit
2. Human PDE6 beta subunit

K

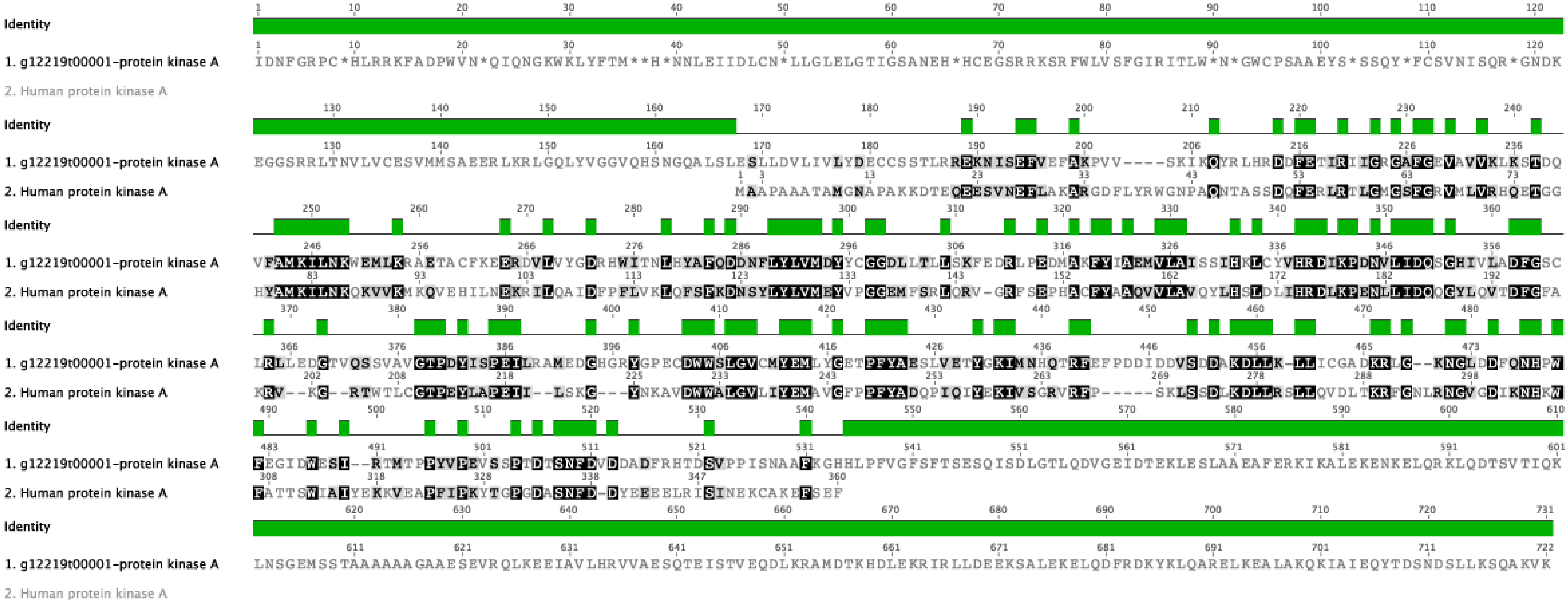
Identity
1. g12219t00001-protein kinase A
2. Human protein kinase A

L

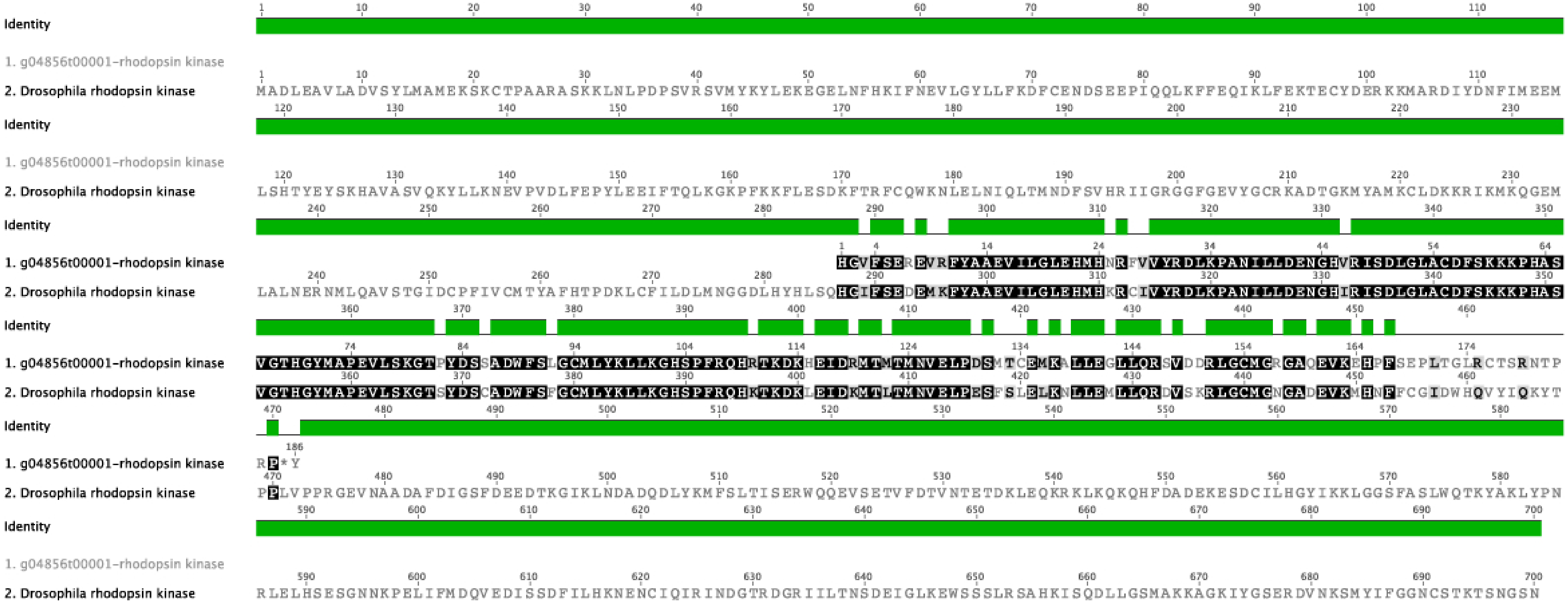
Identity
1. g04856t00001-rhodopsin kinase
2. Drosophila rhodopsin kinase
