# Supplementary material for: De Novo Assembly and Characterization of Two Transcriptomes Reveal Multiple Light-Mediated Functions in the Scallop Eye (Bivalvia: Pectinidae)
Source: PLoS One. 2013 Jul 29;8(7):e69852. doi: 10.1371/journal.pone.0069852 (PMC3726758; doi:10.1371/journal.pone.0069852)

Supplemental Figure 3. Amino acid alignments of scallop circadian clock genes to known homologs. Circadian clock gene sequences from the *Placopecten magellanicus* adult eye transcriptome were translated and aligned to known homologs from *Drosophila*, mouse, or *Crassostrea gigas*. Alignments were completed and exported from Geneious v. 5.6 (www.geneious.com). The graph above the alignment represents mean pairwise identity of each residue pair in each column, with green bars representing identical residues and missing bars highlighting areas where the sequence is different. End gaps in the alignments have solid green bars. Amino acids are shaded based on similarity, with black representing identical residues between the two sequences, grey representing similar residues, and white representing dissimilar residues. *A=clock, B=cryptochrome, C=cycle, D=doubletime, E=period, F=timeless.*

A

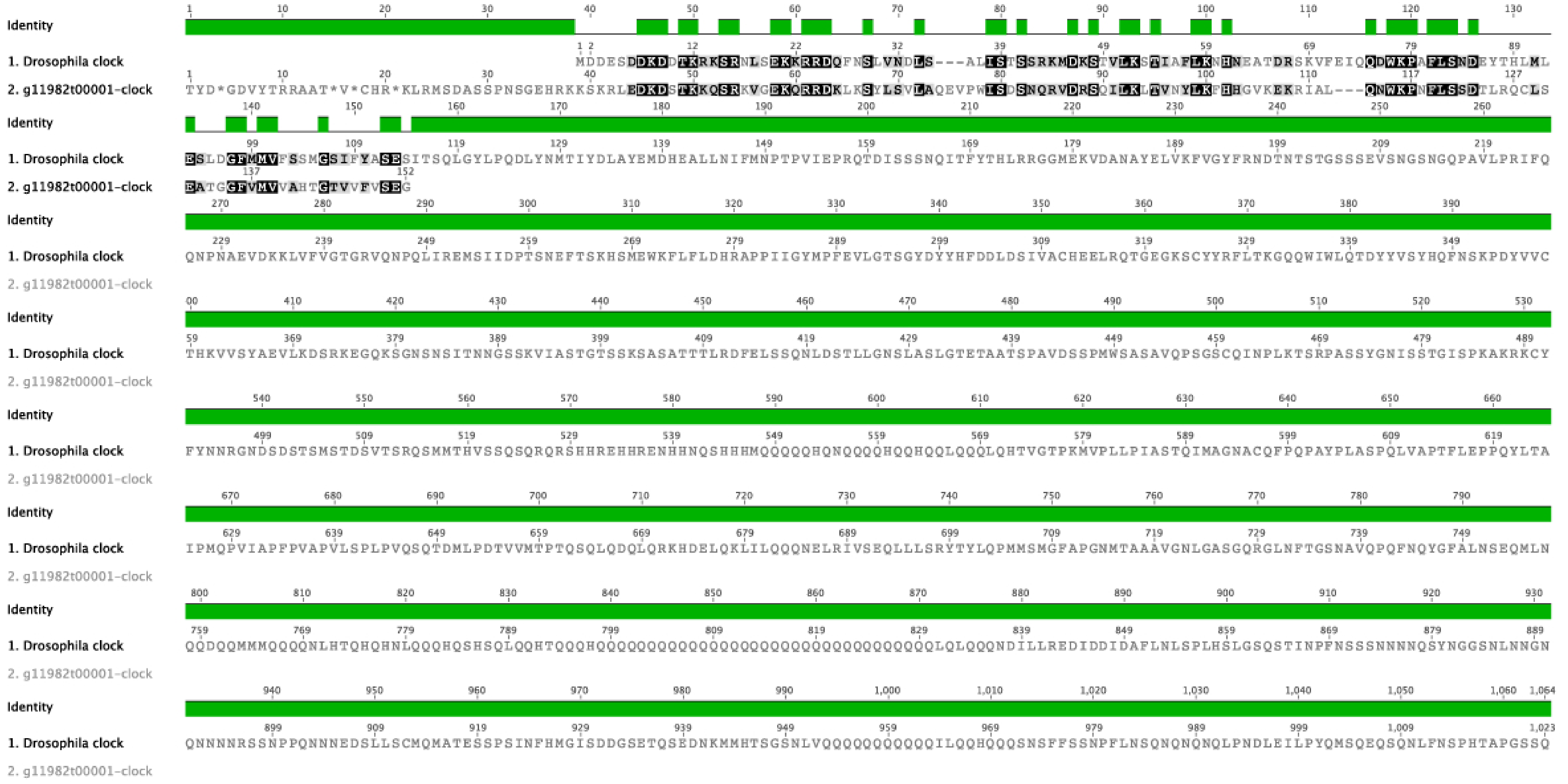

B

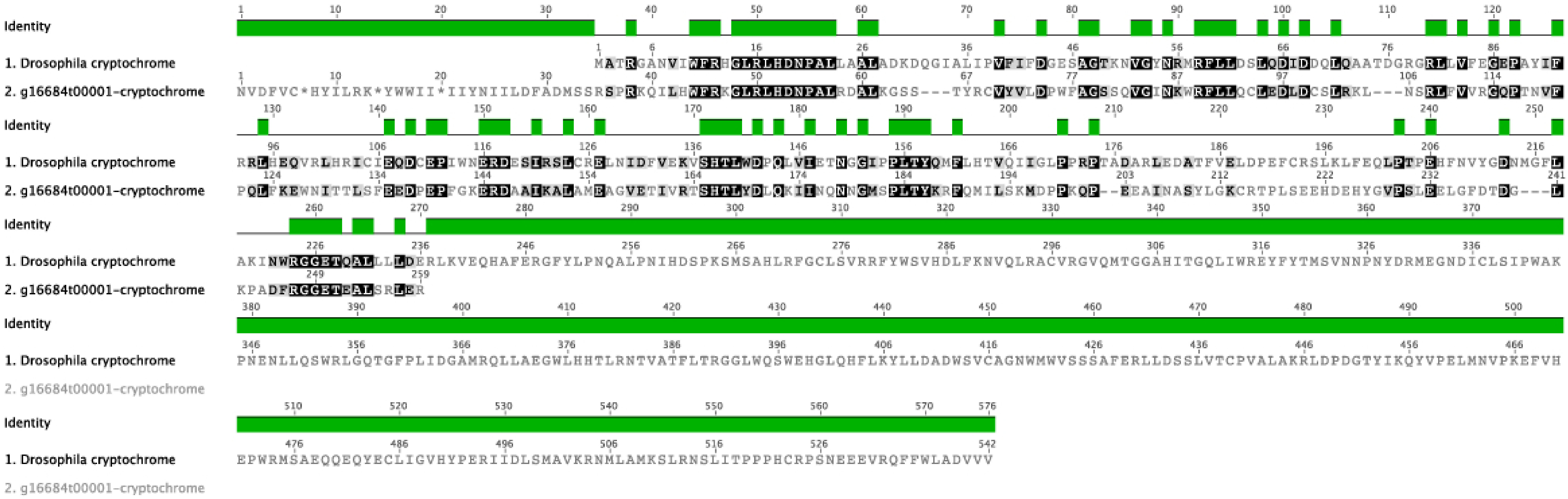

Identity
1. Drosophila cryptochrome
2. g16684t00001-cryptochrome

C

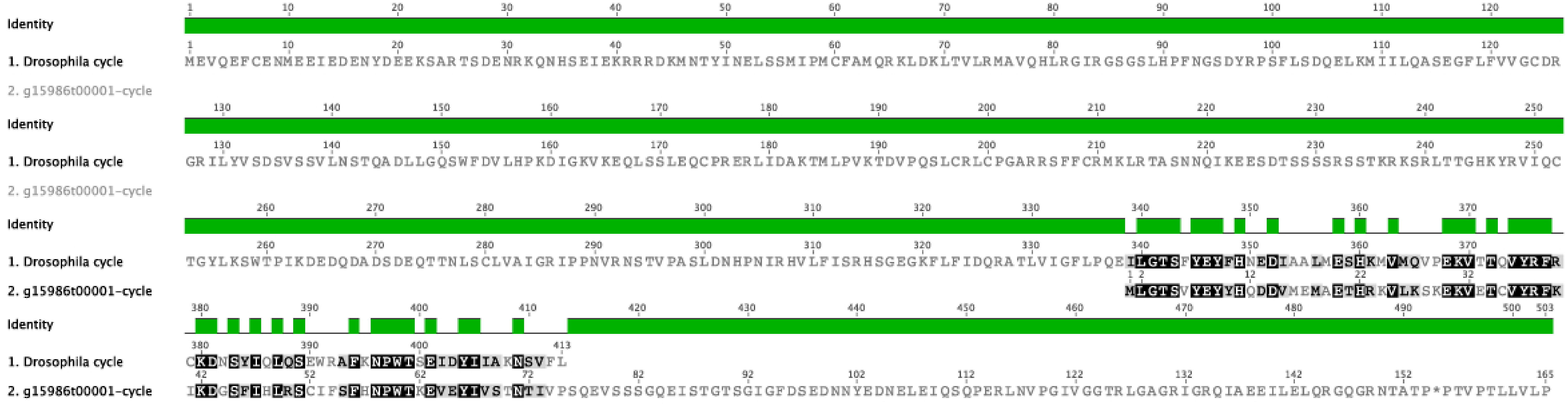

Identity
1. Drosophila cycle
MEVQEFCENMEEIEDENYDEEKSARTSDENRKQNHSEIEKRRRDKMNTYINELSSMIPMCFAMQRKLDKLTVLRMAVQHLRGIRGSGSLHPFNGSDYRPSFLSDQELKMIILQASEGFLFVVGCDR
2. g15986t00001-cycle
Identity
1. Drosophila cycle
GRILYVSDSVSSVLNSTQADLLGQSWFDVLHPKDIGKVKEQLSSLEQCPRERLIDAKTMLPVKTDVPQSLCRLCPGARRSFFCRMKLRTASNNQIKEESDTSSSSRSSTKRKSRLTTGHKYRVIQC
2. g15986t00001-cycle
Identity
1. Drosophila cycle
TGYLKSWTPIKDEDQDADSDEQTTNLSCLVAIGRIPPNVRNSTVPASLDNHPNIRHVLFISRHSGEGKFLFIDQRATLVIGFLPQEILGTSFYEYFHNEDIAALMESHKMVMQVPEKVTQVYRFR
2. g15986t00001-cycle
MLGTSVYEYYHQDDVMEMAETHRKVLKSKEKVECVYRFK
Identity
1. Drosophila cycle
CKDNSYIQLQSEWRAFKNPWTSEIDYIIAKNSVFL
2. g15986t00001-cycle
IKDGSFIHLRSCIFSFHNPWTKEVEYIVSTNTIVPSQEVSSSGQEISTGTSGIGFDSEDNNYEDNELEIQSQPERLNVPGIVGGTRLGAGRIGRQIAEEILELQRGQGRNTATP*PTVPTLLVLP

D

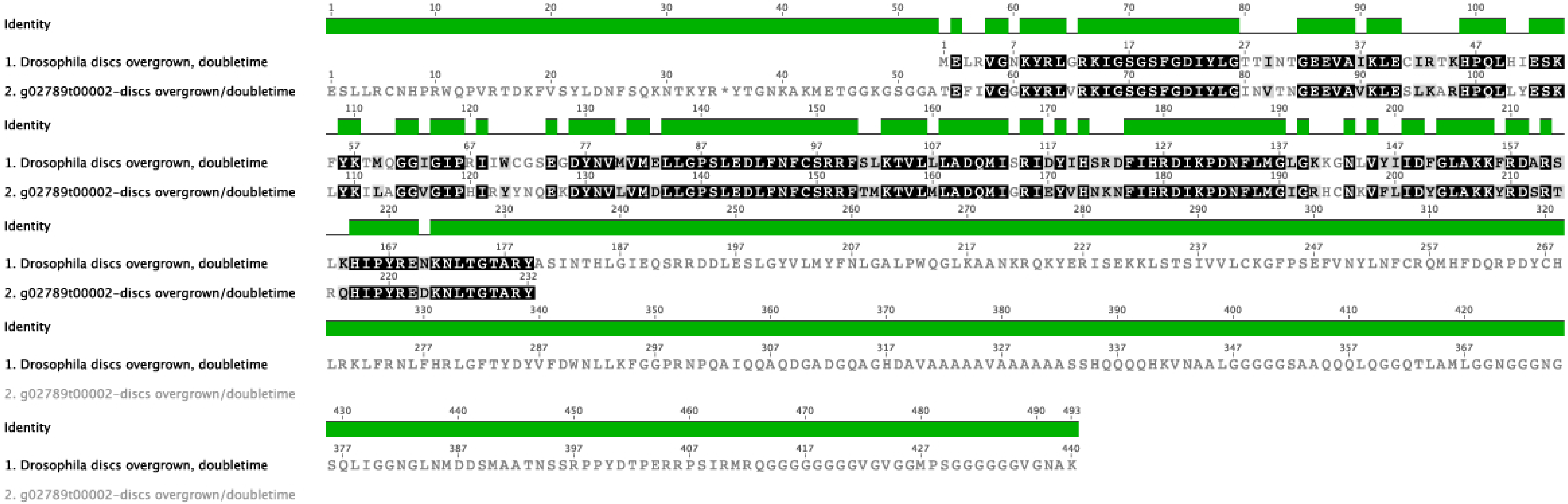
Identity
1. Drosophila discs overgrown, doubletime
MELRVGNKYRLGRKIGSGSFGDIYLGTTINTGEEVAIKLECIRTKHPQLHIESK
2. g02789t00002-discs overgrown/doubletime
ESLLRCNHPRWQPVRTDKFVSYLDNFSQKNTKYR*YTGNKAKMETGGKGSGGATEFIVGGKYRLVRKIGSGSFGDIYLGINVTNGEEVAVKLESLKARHPQLLYESK
Identity
1. Drosophila discs overgrown, doubletime
FYKTMQGGIGIPRIIWCGSEGDYNVMVMELLGPSLEDLFNFCSRRFSLKTVLLLADQMISRIDYIHSRDFIHRDIKPDNFLMGLGKKGNLVYIIDFGLAKKFRDARS
2. g02789t00002-discs overgrown/doubletime
LYKILAGGVGIPHIRYYNQEKDYNVLVMDLLGPSLEDLFNFCSRRFTMKTVLMLADQMIGRIEYVHNKNFIHRDIKPDNFLMGIGRHCNKVFLIDYGLAKKYRDSRT
Identity
1. Drosophila discs overgrown, doubletime
LKHIPYRENKNLTGTARYASINTHLGIEQSRRDDLESLGYVLMYFNLGALPWQGLKAANKRQKYERISEKKLSTSIVVLCKGFPSEFVNYLNFCRQMHFDQRPDYCH
2. g02789t00002-discs overgrown/doubletime
RQHIPYREDKNLTGTARY
Identity
1. Drosophila discs overgrown, doubletime
LRKLFRNLFHRLGFTYDYVFDWNLLKFGGPRNPQAIQQAQDGADGQAGHDAVAAAAAVAAAAAASSHQQQQHKVNAALGGGGGSAAQQQLQGGQTLAMLGGNGGGNG
2. g02789t00002-discs overgrown/doubletime
Identity
1. Drosophila discs overgrown, doubletime
SQLIGGNGLNMDDSMAATNSSRPPYDTPERRPSIRMRQGGGGGGGGVGVGGMPSGGGGGGVGNAK
2. g02789t00002-discs overgrown/doubletime

E

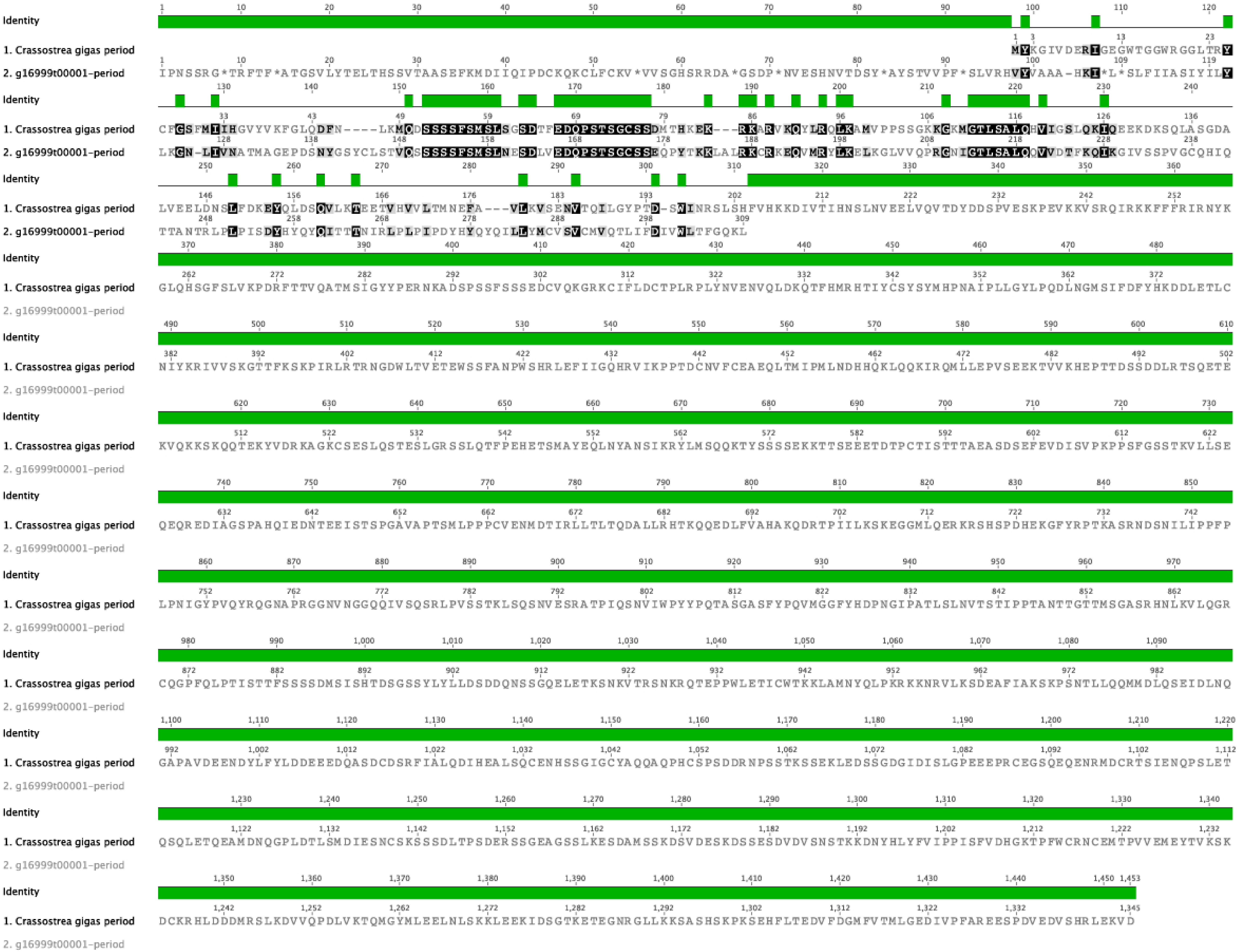
Identity
1. Crassostrea gigas period
2. g16999t00001-period

F

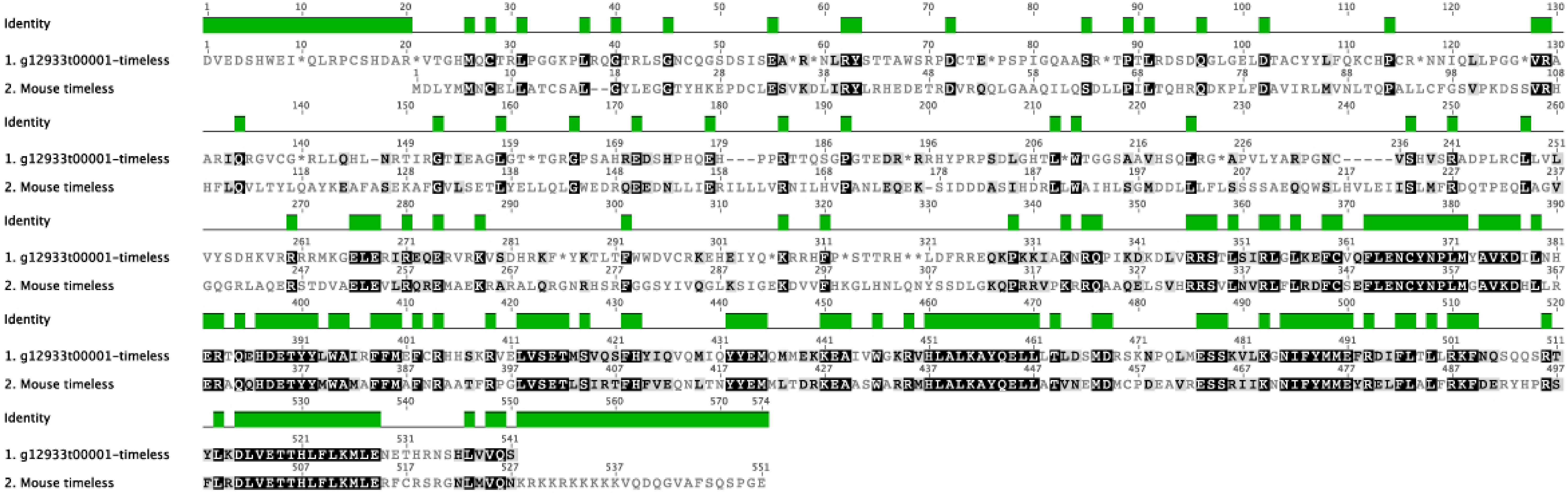

Supplement: Figure S3 — Amino acid alignments of scallop circadian clock genes to known homologs. Circadian clock gene sequences from the Placopecten magellanicus adult eye transcriptome were translated and aligned to known homologs from Drosophila, mouse, or Crassostrea gigas. Alignments were completed and exported from Geneious v. 5.6 (www.geneious.com). The graph above the alignment represents mean pairwise identity of each residue pair in each column, with green bars representing identical residues and missing bars highlighting areas where the sequences differ. End gaps in the alignments have solid green bars. Amino acids are shaded based on similarity, with black representing identical residues between the two sequences, grey representing similar residues, and white representing dissimilar residues. A = clock, B = cryptochrome, C = cycle, D = doubletime, E = period, F = timeless. (PDF) [file pone.0069852.s003.pdf]
